# Plant-Based Scaffolds for Tissue Engineering: A Review

**DOI:** 10.3390/polym17192705

**Published:** 2025-10-08

**Authors:** Maria Isabela Vargas-Ovalle, Christian Demitri, Marta Madaghiele

**Affiliations:** 1 Department of Engineering for Innovation, University of Salento, 73100 Lecce, Italy; 2 Department of Experimental Medicine (DiMeS), University of Salento, 73100 Lecce, Italy; christian.demitri@unisalento.it

**Keywords:** plant-based scaffolds, decellularization, recellularization, tissue engineering, porosity, mechanical properties

## Abstract

The global need for tissue and organ transplantation paved the way for plant-based scaffolds as cheap, ethical, and valuable alternatives to synthetic and animal-derived matrices for tissue regeneration. Over the years, the field has outgrown its initial scope, including the development of tissue models, platforms for drug testing and delivery, biosensors, and laboratory-grown meat. In this scoping review, we aimed to shed light on the frequency of the use of different plant matrices, the main techniques for decellularization, the functionalization methods for stimulating mammalian cell attachment, and the main results. To that purpose, we searched the keywords “decellularized” AND “scaffold” AND (“plant” OR “vegetable”) in online-available databases (Science Direct, Scopus, PubMed, and Sage Journals). From the selection and study of 71 articles, we observed a multitude of plant sources and tissues, along with a large and inhomogeneous body of protocols used for decellularization, functionalization and recellularization of plant matrices, which all led to variable results, with different extents of success (mostly in vitro). Since the field of plant-based scaffolds shows high potential for growth in the next few years, driven by emerging biotechnological applications, we conclude that future research should focus on plant sources with low economic and environmental impacts while also pursuing the standardization of the methods involved and a much deeper characterization of the scaffold performance in vivo.

## 1. Introduction

According to the 2023 International Report by the Global Observatory on Donation and Transplantation (GODT), produced in collaboration with the World Health Organization (WHO) and Observatorio Nacional de Transplantes (ONT)—Spain, more than 157,000 solid organ transplants were performed globally in 2022, covering less than 10% of the total global demand (CIT, data from 2022) [1]. This figure only accounts for solid organ transplants; when considering the need for other human tissues and cells, the estimated shortfall grows significantly, highlighting the persistent gap between supply and demand [1].

Despite various recent campaigns aimed at increasing the availability of cells, tissues, and organs for transplantation, demand continues to outstrip supply, also due to limitations in tissue storage and transportation [2].

In parallel, basic and translational research increasingly needs reliable in vitro models of diverse and specialized tissues to replicate the in vivo environment more closely. Scalable, biodegradable, and biocompatible scaffolds—both for clinical use and scientific research—hold promise in addressing these challenges [3].

Tissue-engineering strategies exploit a particular toolbox to create tissues and organs, which consists of the following: (1) the cells that make up the tissue, (2) tissue-inducing soluble molecules or mechanical regulators, and (3) the matrix or scaffold that facilitates the cellular interactions and instructs the cells to form the desired tissue [4].

In this review, we particularly focus on the growing roles of plant-based scaffolds in tissue-engineering and biomedical research, as well as in the production of laboratory-grown meat. The unique microarchitecture of plants and vegetables, together with their biocompatibility and wide availability, make decellularized plant matrices an intriguing option for tissue engineering. In the following, we briefly discuss the rationale for using plant-based scaffolds and the related challenges, which are then addressed in this work.

### Why Plants?

Scaffolds for tissue engineering are commonly synthesized starting from various biodegradable and biocompatible biomaterials (of synthetic and/or natural origin(s)) [5], which are then processed to obtain a given microstructure, mimicking the architecture of the target tissue. An alternative and well-established approach to obtain the scaffolds is to directly borrow the composition and the architecture of tissues: In other words, animal tissues are decellularized, and the resulting extracellular matrix is then used as a scaffold for cell growth [6]. In any case, the scaffold, either synthetic or a decellularized matrix, is meant to directly interact with mammalian cells and guide the tissue formation [7].

Plants and vegetables also have their own extracellular matrix, mostly composed of cellulose and hemicellulose, along with pectin and lignin (with the exact composition varying across species and being influenced by environmental factors) [8,9]. Cellulose shows high biocompatibility and carries low immunogenicity [10,11,12]. However, it cannot interact with mammalian cells and is poorly degradable in the physiological environment [9,13,14]. This is probably why the idea of using decellularized plant and vegetable matrices as potential scaffolds seemed to be ignored by researchers at the beginning of tissue engineering, with a pioneering work by Modulevsky et al. only appearing in 2014 [15]. However, the field has, since then, significantly expanded, suggesting that plant-based matrices offer a competitive and promising alternative to both synthetic and animal-derived ones [16].

First, one of the distinctive advantages of plant-based scaffolds is their naturally occurring vasculature and porous microstructure [13]. These features, essential for tissue engineering, facilitate the distribution of nutrients and the removal of waste, both of which are critical for supporting mammalian cell growth, especially in high volumes. Unlike synthetic scaffolds, which often struggle to achieve a continuous and somewhat uniform porosity, plant-derived scaffolds inherently possess a well-organized architecture that is optimized for fluid transport and cellular interactions [17]. The naturally pre-existing structure of plant scaffolds, including their interconnected pores and vascular networks, is difficult to replicate through synthetic scaffold preparation techniques, whether using top-down or bottom-up approaches [16]. In synthetic scaffolds, achieving the same balance between structural integrity and porosity often involves complex and costly manufacturing processes, which still may not result in ideal scaffolds for tissue growth. This inherent advantage of plant-derived matrices makes them a biocompatible and cost-effective alternative to synthetic scaffolds for tissue-engineering applications [18,19,20,21].

Moreover, when compared to animal-derived matrices, plant- and vegetable-based ones offer a more ethical approach to tissue regeneration, independent of animal exploitation and free from any cultural or religious concerns. Notably, they also represent a generally safer alternative for clinical use, carrying no risk of zoonosis [22,23].

Finally, the abundance and large variety of plants and vegetables hold promise for the development of a multitude of cost-effective scaffolds with potential for the regeneration of diverse and/or complex tissues. Therefore, it is not surprising that plant-based scaffolds have attracted growing interest over the last 10 years.

However, as previously mentioned, the use of plant-based matrices for tissue regeneration also carries inherent challenges. First, the optimization and standardization of plant decellularization protocols is fundamental to achieve matrices with reproducible properties and in which the pre-existing vasculature, porosity, and mechanical resistance of the plant cell walls are preserved as much as possible [18]. Agents or processes used for the decellularization should also be cytocompatible and safe for tissue-engineering applications. While the impact(s) of the decellularization protocol(s) on the scaffold properties appear(s) obvious, there can be large variability in protocols used by different researchers and/or for different plants, which makes it very difficult to identify optimal and standard protocols, if any [23].

A further, and likely more complex, challenge is then represented by the recellularization of the scaffolds, as cellulose lacks attachment sites for mammalian cells or extracellular matrix proteins, thus requiring surface functionalization and/or proper cell-seeding strategies to promote and facilitate the interactions with cells [24]. Also, in this regard, various approaches can be tested by researchers, with different extents of success; this also depends on the cell type(s) and the target tissue to regenerate, which adds a further layer of complexity [25].

The poor degradability of cellulose is another intrinsic factor that could challenge the successful use of plant-based scaffolds for tissue engineering and should be carefully assessed in scaffold characterization. In this matter, it is also interesting to observe that plant-based, edible scaffolds (even if poorly degradable) may still find use in the production of cultivated meat, where scaffold residues could even contribute to the taste, texture, and nutritional profile of the cultivated meat [26,27].

Based on the increasing scientific and technological interest in plant-based scaffolds, the challenges faced for their successful application, and the inhomogeneous body of protocols and tests across studies, a scoping review was conducted to systematically map the research conducted in this area (with a special focus on decellularization, recellularization, and performance), as well as to identify potential gaps in knowledge. To this purpose, the following research question was formulated: “What is known from the literature about decellularized plant- or vegetable-based scaffolds, the decellularization techniques to obtain them, the methods to enhance recellularization, and their biological performances and potential applications?”

## 2. Methodology

### 2.1. Protocol

This research was performed with reference to the Preferred Reporting Items for Systematic Review and Meta-Analysis extension for scoping reviews (PRISMA-ScR) guidelines [28].

### 2.2. Eligibility Criteria

Research articles were eligible for the analysis if written in English, published between 1989 and March 2025, and dealing with a plant/vegetable tissue (stem, leaf, hypanthium, or root) that was decellularized for subsequent recellularization with cells of mammalian origin.

To perform a broader and deeper analysis of the field, articles where the plant matrix suffered a physical change upon decellularization, such as being pulverized or mechanically disrupted, were also eligible, as well as articles focusing on non-plant matrices, such as algae and fungi; compared to plant matrices, the latter, indeed, share similar applied protocols and fields of application.

Only full-text articles with open access or full institutional access were included. Abstract publications only, preprints, letters without primary data, review articles, comments, and theses were excluded.

### 2.3. Information Sources and Search

A systematic literature search was conducted in Science Direct, Scopus, PubMed, and Sage Journals (as summarized in Table 1), with the following combined keywords: “decellularized” AND “scaffold” AND (“plant” OR “vegetable”). Where possible, further filters were applied, such as “Article type > Research articles” and “Languages > English”, and the search results were exported in .csv format.

The entire search was performed between January 2024 and April 2025, with the most recent analysis of the databases executed on 29 April 2025.

### 2.4. Selection of Sources of Evidence and Data Charting

First, the titles and abstracts of the articles obtained by the systematic search were analyzed. If fulfilling the eligibility criteria, the full text articles were reviewed to assess their actual relevance to the topic.

If relevant, the entries were included in a new form elaborated by the authors, where the following information was collected: (a) first author, (b) full authors, (c) year of publication, (d) title, (e) abstract, (f) DOI, (g) type of matrix, (h) decellularization technique, (i) type of cellular study, (j) strategy for cell adhesion, and (k) primary outcome or conclusion. The information that was added to the form was verified at each new entry and upon a final check of all the entries.

When found, any duplicates were eliminated. The references cited in the articles were also reviewed to identify any relevant study potentially missing in the electronic database.

Information from the final article selection was analyzed and grouped to obtain relevant data for the research question under investigation, like the used matrix, decellularization principle and protocol, adhesion moiety or functionalization, cell line or cell type, potential application, and main results.

## 3. Results

### 3.1. Article Selection

**Figure 1:**
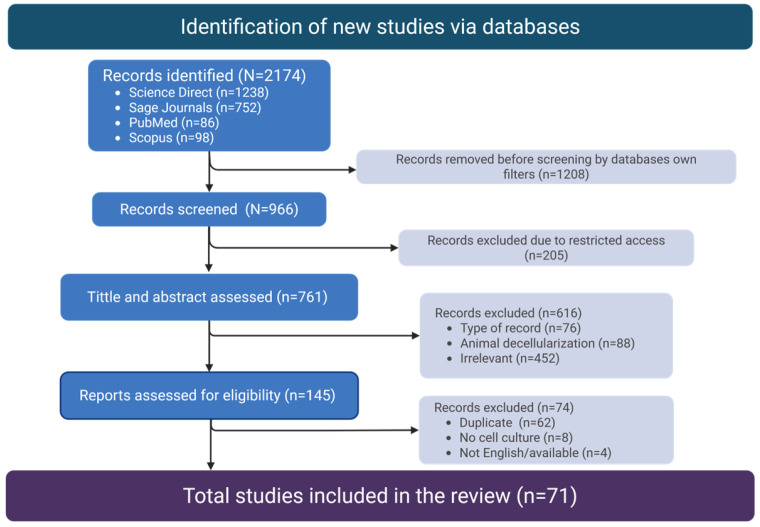
PRISMA flowchart for the study selection.

Figure 1 shows the roadmap of the study selection process. The initial total number of identified studies was 2174, which decreased to 966 after using appropriate website filters to select research articles only. After this step, a further 205 studies were excluded because of the lack of institutional access. The remaining 761 sources were then screened for title and abstract. A further 616 studies were thus excluded because they were not relevant to the research question under investigation, being different from research articles (*n* = 76) dealing with decellularization of mammalian tissues (*n* = 88) or including plant tissues but for applications beyond the scope of this review (*n* = 452). At this point, the full text of the remaining one hundred forty-five articles was evaluated, of which sixty-two were duplicates, eight were not dealing with the culture of mammalian cells, and four were not available in the English language. One additional article was included, even though it did not appear in the search results indicated before, yet it was consistently cited by many of the selected sources. Finally, a total of 71 research articles were found to meet the predefined criteria and were included in this review.

### 3.2. Characteristics of Sources of Evidence

A summary of the main characteristics of the literature references analyzed in this study is provided in Table 2, which details the plant matrix and the decellularization protocol(s) assessed in each study, together with the main biological tests and findings. Further information on enhancing cell adhesion strategies and matrix characterization is provided in the following sections.

### 3.3. Synthesis of Results

**Figure 2:**
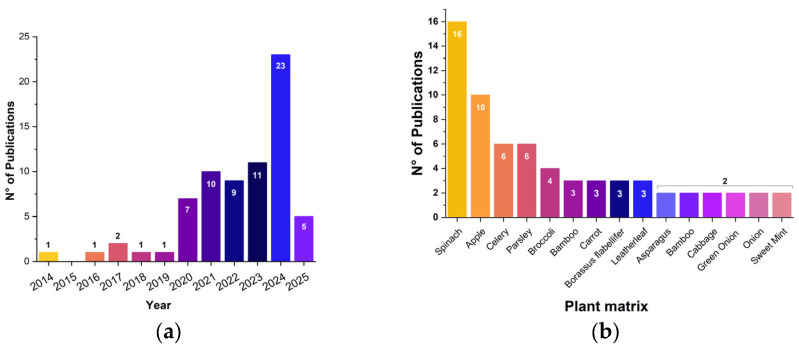
Growing interest in plant-based scaffolds derived from different plants. (**a**) Number of related publications over the years; (**b**) most used plant sources reported so far.

A first glance at the study selection evidenced a three-fold increase in the number of publications in the last 4-year period, from 2020 to 2024 (Figure 2a). This confirmed the increasingly growing research interest in plant-based scaffolds. As for the number of articles in 2025, it should be recalled that this number does not include articles published after April 2025; thus, it is highly likely to increase by the end of the year.

Regarding the main results obtained from the analysis of the sources of evidence, the following synthesis should be taken with the premise that some of the selected articles tested more than one plant matrix [30,31,32,33,34,35,37,44,53,63,65,73,97,98,99], more than one decellularization protocol [32,41,44,64,67,73,79,94], or more than one adhesion strategy at the same time or as a part of a comparison or standardization study [36,38,52,54,56,57,63,66,69,79,89,91,97,98,99]. Therefore, the total number of plant matrices or used protocols reported in the following may be higher than 71.

#### 3.3.1. Selection of the Plant Matrix

It was noticed that the most frequently used plant matrices (Figure 2b) were spinach or spinach leaves [31,36,38,39,44,45,46,54,63,64,76,77,84,88,96,97] in sixteen studies, followed by apple [15,29,33,34,35,55,66,83,86,91] in ten, celery [34,35,44,78,81] and parsley [30,31,44,63,74,75] in six, broccoli [33,34,43,59] in four, and carrot [33,34,35], leatherleaf [63,79,94], and Borassus flabellifer [47,57,58] in three. Asparagus [34,61], bamboo [30,41], cabbage [49,50], green onion [34,97], onion [48,99], sweet mint [44,54], tomato leaves [54,71], and species of *Anthurium* sp. [30] were all used two times. Additionally, 60 plant matrices were reported only once.

#### 3.3.2. Decellularization Protocol

**Figure 3:**
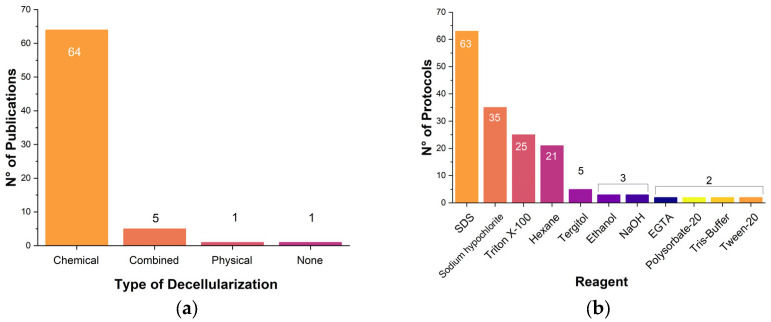
Summary of the reported decellularization protocols. (**a**) Technique or principle used for the decellularization of the plant matrices; (**b**) overview and frequency of use of the main types of surfactants adopted in the case of chemical decellularization.

In the analyzed 71 studies, a total of 89 procedures were reported for decellularization, depending on the plant source and tissue, and differing for the type of approach (chemical, physical, or combined) and chemicals/agents used. The chemical approach was used in most cases (Figure 3a). Looking more closely at the chemical protocols (Figure 3b), the majority (i.e., 63 protocols) involved the use of sodium dodecyl sulfate (SDS), also in combination with different chemicals. Among other reagents, sodium hypochlorite was reported in 35 protocols, Triton X-100 in 25 protocols, and hexane in 21 protocols. It was also found that Tergitol was used in five protocols [63,94], and ethanol [61,81,88] was used in three studies, like NaOH [69,89,90]; EGTA was evaluated in two protocols [63,94], and TRIS-HCl buffer [38,90] and Tween-20 [59,84] were used in two protocols as well. Other commonly found chemicals cited at least once included acetic acid, acetone, and bleach.

Even though most of the protocols included SDS and Triton X-100, it should be noted that both surfactants have a very low LD50 dose (Table 3), suggesting that any residues of these chemicals in decellularized matrices may have harmful, toxic effects on cell cultures or animal models. Therefore, researchers have often explored alternative surfactants and/or tried to reduce their quantities by coupling them with safer chemicals, such as those listed in Table 3. In any case, one of the challenges presented by plant matrix decellularization is the careful removal of the chemicals used in the final scaffold. It is also clear that the search for friendlier chemicals should continue, especially when thinking of the potential use of plant-based scaffolds for clinical applications or for the production of cultivated meat. With reference to the latter case, some authors have recently tried with the use of polysorbate-20, an authorized agent commonly adopted in the pharmaceutical, cosmetic, and food industries [64,65].

In addition to chemical methods, other decellularization protocols found in the literature involved the utilization of mixed chemical and biological means, for example, decellularization with trypsin [94] or lysozyme [56]; physical methods could be also found, as in the case of lyophilization and DNase in the treatment of rice cells and tobacco cells and tissues [37]. Another approach was the use of supercritical CO_2_ for the decellularization of spinach, sweet mint, celery, and parsley [101]. In addition to the aforementioned techniques, one report indicated the use of a mixed chemical (NaOH) and physical treatment (temperature) to denature leaf veins [87]; another one mentioned the use of SDS, sodium carbonate, sodium chlorate, and increased temperatures to decellularize different algal species [73].

#### 3.3.3. Cell Adhesion Strategy

**Figure 4:**
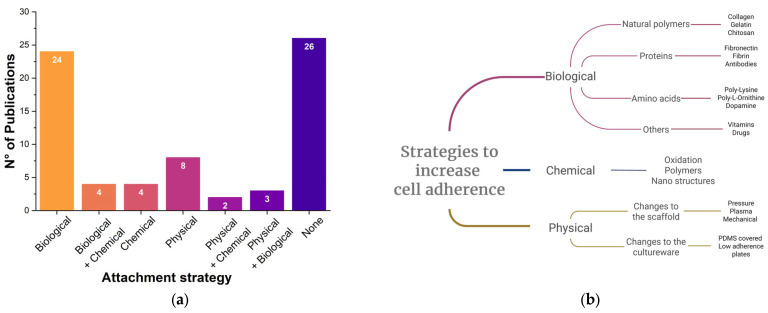
Strategies to enhance cell attachment to plant-based scaffolds. (**a**) Distribution of the main principle/strategy adopted in the selected studies and (**b**) summary of the biological, chemical, and/or physical methods used to increase the success of mammalian cellular attachment to the scaffolds.

As commented above, cellulose lacks the attachment epitopes necessary for mammalian cells to adhere; thus, researchers have tried different approaches to stimulate plant matrix recellularization (Figure 4). However, it is worth noting that some studies have had quite successful results, even without the application of any enhancing cell attachment method, with varying results [29,37,39,40,43,48,49,55,59,60,65,67,68,70,73,74,81,83,84,85,87,88,90,94,95,96]. The nonuse of scaffold functionalization is not per se a negative aspect, since plants’ physical cues, such as natural topography and stiffness, could suffice to stimulate the invasion, homing, and proliferation of the cells. For example, Triton X-100-decellularized scaffolds based on olive leaves allowed the growth of C619 cells (when Triton X-100 was used at low concentrations) [67], while Nopal scaffolds could be successfully seeded with hDP-SCs [70]. In the case of decellularized *Bougainvillea* sp. flowers tested with NIH-3T3 and HaCaT, the natural grooved surface of the plant behaved as a suitable biophysical cue for cell adhesion [90].

When talking about the functionalization strategies explored by researchers, a large variety of adhesion-enhancing moieties have been tested so far for deposition on the scaffold surface, including polymers naturally found in the ECM [68], such as collagen [15,36,97], its denatured version (gelatin) [63,75,77,79,91], proteins (like fibronectin [31,34,36,38,54,79] and fibrin) [50], other natural polymers (such as chitosan) [47,52,57], some extracts (like platelet-rich plasma (PRP) [57] or grape-seed-rich extract) [62,80], various amino acids [35,61,92], antioxidants [76], or conjugated catecholamines [30,32,45,72].

Some authors opted for a chemical modification of the scaffold, e.g., using sodium periodate for oxidation [41] or amino (NH_2_)-terminated 3-aminopropyltriethoxysilane (APTES) or methyl (CH_3_)-terminated octadecyltrichlorosilane (OTS) [58]; nanoamyloids and nanohydroxyapatite [56] were also reported. Other more complex biochemical adhesion strategies included the use of a poly(PEGMEMA-*r*-VDM-*r*-GMA) copolymer modified with the RGD peptide [69], polyaniline and GO [89], copper ion/gallic acid MOFs via a PVA-TSPBA hydrogel [93], and magnesium oxide particles [82].

Regarding the physical methods that may be used to induce or facilitate cellular attachment to plant-based scaffolds, it is interesting to observe that several studies aimed at modifying the surface of the cell culture plates, instead of the scaffold one, by increasing their hydrophobicity (e.g., via the use of a PDMS coating): This strategy may effectively drive or force the attachment of mammalian cells to vegetable-derived scaffolds. For example, this approach has been used for scaffolds made from apple [91], carrot and persimmon [33], as well as spinach [64,97] and celery [78]. Other physical stimulation strategies for affecting cell attachment have involved the use of cyclic hydrostatic pressure [55] or plasma to render the scaffold surface more hydrophilic [60,71]. Finally, the use of negative pressure to infuse the scaffold with probiotic cells has also been reported [66].

#### 3.3.4. Additional Tests

The selected sources of evidence suggested that a key point to consider for the successful use of plant-based scaffolds in the biotechnological context is their mechanical performance. Some of the tested parameters and results are summarized in Table 4, yet for a full disclosure of the calculating methods, end points, maximum deformation, the samples’ dimensions, or the number of scaffolds tested, it is advised to visit the original sources.

**Figure 5:**
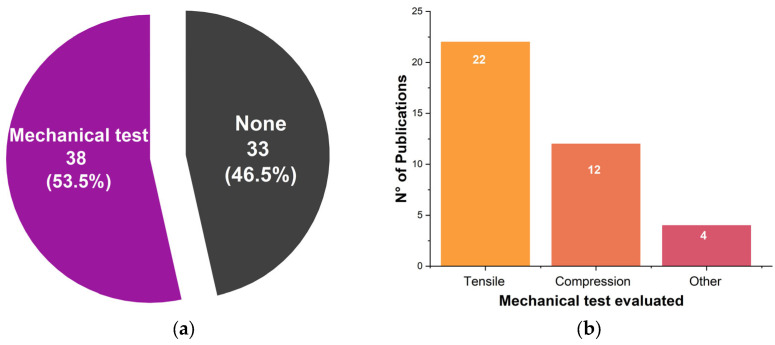
Importance of mechanical tests in the characterization of plant-based scaffolds. (**a**) Pie chart indicating the distribution of publications that assessed the mechanical behavior of the scaffold; (**b**) insight into the different mechanical tests being reported.

Since one of the potential applications of plant-based scaffolds is the production of native tissues in vitro or in vivo, more than half of the studies (Figure 5a) tested their scaffolds in terms of either compressive and tensile strengths or suture retention properties (Figure 5b) or all of them [93]. For reference, the elastic modulus of healthy adipose tissue is 2–3 kPa, while for tumor tissue in the first stages of development (6–10 kPa) [102], muscle (12–38 kPa), fat (91–109 kPa), skin (330–1.28 × 10^3^ kPa) [103], and bone (5.7–17.9 kPa) [104]. 

**Table 4:** Summary of mechanical tests divided by reference, plant matrix, type of test, setting parameters, and main results. Details regarding the test conditions, specific temperatures, dimensions, and equipment can be consulted in each primary source.

Plant Matrix	Mechanical Test			Ref.
	Type	Parameters	Main Results	
Spinach Parsley *Artemisia annua* Peanut hairy leaves	Tensile	Native and decellularized spinach leaves were uniaxially stretched at a constant strain of 10 mm/min until failure.	Decellularized leaves displayed significantly lower ultimate tensile strength (*p* = 0.00925) and strain at failure (*p* = 0.000287) than native samples. Maximum tangent modulus for decellularized spinach leaves was 0.3 MPa.	[31]
*Ficus hispida* *Paquira aquatica* *Garcinia* sp.	Tensile	Maximum tangent modulus (MTM), strain at failure (SAF), and ultimate tensile strength (UTS) for *F. hispida* and *Pachira aquatica* samples.	*P. aquatica* samples prepared using both decellularization protocols displayed similar mechanical properties across all three of the parameters measured. The *F. hispida* samples showed a similar trend in all the cases except the UTS testing. Samples prepared using the SDS had higher average UTS results than those prepared with bleach.	[32]
Apple Carrot Celery	Hysteresis compression and tensile	Apple: hysteresis compression cycle. Each test consisted of a load phase at a rate of 2.5% min^−1^ down to −30% strain. Carrot: compression load applied at a rate of 5%/min up to 60% strain. Celery: tensile load applied at a rate of 20% strain.	After the different tests, these are the elastic moduli of the different matrices: E apple = 4 kPa, E carrot = 43 kPa, E celery = 590 kPa.	[35]
Spinach	Tensile	Tensile test at a loading velocity of 5 mm/min.	The peak of stress–strain curve was 1.4 MPa and its elongation at break 4.57%.	[39]
*Bambusa vulgaris*	Compression	Uniaxial compression down to 80% compression at a crosshead speed of 5 mm/min.	The decellularized bamboo had a compressive strength of 1.52 ± 0.346 MPa, whereas the values of the ones oxidized (ODP 0.01, ODP 0.1 and ODP 0.5) were 1.36 ± 0.47 MPa, 1.078 ± 0.2 MPa and 0.6 ± 0.045 MPa, respectively. Thus, the strength was decreased, in correlation with the oxidation process (DP > ODP0.01 > ODP0.1 > ODP0.5).	[41]
Chive Spinach	Tensile	Uniaxial tensile loading until failure.	It was not possible to apply traverse tensile loading on the chive for mechanical testing. Only tensile loading in the longitudinal direction along the cellulose fibers. When possible, results show stress at break was 0.7 ± 0.2 MPa, and the strain at break was 20 ± 13% compared with 13 ± 3% in native tissue.	[45]
*Borassus flabellifer*	Compression	Hydrated scaffold samples compressed at a rate of 1 mm/min.	The stress–strain curve is typical for soft polymers with the stress plateau between 60 and 70% of strain. The compressive strength was found to be higher in cellulose–chitosan group.	[47]
Onion	Tensile	Tensile test with a loading speed of 5 mm/min.	The results demonstrated that scaffold revealed a high tensile strength (8.197 MPa), and an elongation at break (3.23%) close to the normal bone tissue.	[48]
Cabbage	Tensile	Cut samples subjected to a loading velocity of 50 mm/min.	Peak of stress-strain curve was 4.32 MPa and its elongation at break was 18.54%.	[49]
*Alstroemeria* flower	Compression	Each stem was placed between the grips of the machine and compressed in the axial direction at the rate of 1 mm/min.	The decellularized scaffold showed an approximately 40% increase in length, while this factor was about 80% for the decellularized and chitosan ones. In terms of the Young’s modulus, the decellularized scaffolds had (0.15 MPa) versus (0.8 MPa) for the ones coated with chitosan.	[52]
Fennel Wild Fennel Dill	Compression	The test was performed by applying a hysteresis compression cycle on the decellularized samples with GelMA and GelMA scaffolds as controls. A preload force (F = 0.001 N) was applied, followed by a loading phase at a rate of 2.5%/min, down to −30% strain, and an unloading phase at a rate of 5%/min.	A decrease in mechanical characteristics was found for all the samples containing plant structures compared to GelMA samples. GelMA-Fennel (4.27 +/− 1.16 kPa), GelMA-Wild Fennel (5.68 +/− 0.76 kPa), GelMA-Dill (3.55 +/− 0.34 kPa) versus GelMA (9.32 +/− 1.85 kPa).	[53]
Spinach Sweet Mint Tomato leaves	Tensile	PDMS, decellularized spinach and porcine lung tissue were evaluated. The samples’ stretching properties were analyzed in a custom build device. Additionally, the stretched and non-stretched spinach leaves were subjected to a constant strain of 5 mm/min until failure.	The local longitudinal strain values of the PDMS were between 10.85–12.71%) while the decellularized leaf and lung scaffolds showed greater variation, ranging from 7.76–15.88% and 10.67–19.67%, respectively.	[54]
Apple	Compression	Cyclic hydrostatic pressure (HP) stimulation.	Data showed no significant changes between samples incubated in osteogenic media with applied HP (16.1 ± 2.1 kPa) and without applied HP (17.2 ± 3.2 kPa) after 1 week and 2 weeks (13.9 ± 0.8 kPa and 18.7 ± 0.7 kPa, respectively).	[55]
Celery	Tensile	A universal mechanical testing machine was used to measure the Young’s modulus (no more details indicated in the experimental section).	The Young’s modulus values of decellularized celery with nanoamyloids and decellularized celery with nanohydroxyapatite and were significantly higher than that of decellularized celery.	[56]
*Borassus flabellifer*	Compression	Samples under hydrated conditions were evaluated at a rate of 1 mm/min.	All scaffold groups (cellulose, cellulose-chitosan, cellulose-chitosan-PRP and cellulose-PRP) exhibited linear elastic behavior at lower stress values. Up to 80% strain, all the scaffold groups exhibited an extended linear elastic region without plateau or final densification region. The compressive strength was higher among the chitosan coated samples than in the uncoated decellularized ones. The compressive moduli were found to be in the following decreasing order: Cellulose-Chitosan-PRP, Cellulose-Chitosan, Cellulose-PRP and Cellulose. The presence of PRP did not modify the mechanical behavior.	[57]
*Borassus flabellifer*	Compression	Compressive mechanical properties of the scaffolds were estimated under hydrated conditions with a speed of 1 mm/min.	The compressive moduli were found to be 0.59 ± 0.19 kPa for oxidized scaffolds, 5.73 ± 1.76 kPa oxidized + APTES, and 8.33 ± 1.52 kPa for OCS and OTS. Surface modification by APTES and OTS has a significant effect in enhancing the compressive mechanical behavior of OCS scaffolds.	[58]
Asparagus	Compression	Each scaffold was compressed mechanically to a maximum 30% strain, at a compression speed of 50 µm/s.	The Young’s modulus of the scaffold in culture media at 37 °C is 128 ± 20 kPa when measured parallel to the long axis.	[61]
Leatherleaf Spinach Parsley	Tensile	Samples (from the different tissues leatherleaf, spinach, and parsley, and the different decellularization protocols, as well as cross-linked gelatin) were pulled uniaxially at 0.08 mm/s until failure.	Elastic modulus was 3.8 ± 0.2, 4.0 ± 1.4, 3.9 ± 0.3, 2.7 ± 0.9, and 2.8 ± 0.2 MPa for intact, SDS-, SDS/EGTA-, Tergitol/EGTA-, and Tergitol/SDS-decellularized leatherleaf, respectively. Elastic modulus of spinach was 1.15 ± 0.22, 1.62 ± 0.58, 0.54 ± 0.77, 4.05 ± 1.33, and 2.34 ± 1.09 MPa for intact, SDS-, SDS/EGTA-, Tergitol/EGTA-, and Tergitol/SDS-decellularization, respectively. Parsley was 4.47 ± 0.16, 3.59 ± 1.47, 2.28 ± 0.62, 1.21 ± 0.17, and 0.21 ± 0.29 MPa, respectively. Elastic modulus of cross-linked gelatin was 0.3 ± 0.1 MPa, and failure strain was 0.1 ± 0.02. Maximum modulus of 3D grafts constructed from SDS-decellularized leatherleaf and gelatin was 1.3 ± 0.1 MPa. Maximum tensile stress at failure was 5.5 ± 1.1 MPa, and failure strain was 4.1 ± 0.7.	[63]
Corn Husk Jackfruit rind	Compression and tensile	Fresh and decellularized jackfruit and corn husk samples were pulled to failure. Jackfruit samples were axially affixed into the machine and pulled at a constant 10 mm/min. Corn husk samples were pulled to failure both parallel to leaf venation and orthogonal to it.	Following decellularization, corn husk scaffold stiffnesses decreased from 56.67 ± 16.71 MPa to 12.95 ± 2.43 MPa in fiber-aligned direction, while jackfruit decreased from 7.54 ± 2.42 MPa to 2.47 ± 1.47 MPa.	[65]
Apple	Compression (hardness and stiffness)	To measure hardness, samples were compressed to 50% of the initial thickness. Maximum force (N) of compression was measured. To measure stiffness, Young’s modulus (MPa) was calculated based on the force displacement curve.	The hardness of the decellularized apple tissues was reduced 1.5 times from the native ones (33,42 ± 7.62 N) vs. (51.99 ± 7.33 N). The Young’s Modulus on the other hand was reduced from a native one of (0.3 ± 0.09 MPa) to (0.1 ± 0.01 MPa) in decellularized samples.	[66]
Olive Leaves	Tensile	A uniaxial tensile test at constant force at a speed of 10 mm/min was applied, and the sheets were pulled to the rupture point.	The elasticity modulus of acellular samples was significantly reduced compared to normal leaf samples. The no decellularized sample showed a modulus of elasticity of about 20.45 MPa and a maximum tensile stress of 2.50 MPa. While the decellularized samples showed an elasticity modulus of 4.99 MPa and a maximum tensile stress of 0.60 MPa.	[67]
Nopal	Tensile	Native and decellularized nopal samples. A cross speed of 1 mm/min applied until failure occurred.	Native tissue showed a tensile strength of 12.5 +/− 1 MPa and decellularized tissue of 11.8 +/− 0.5 MPa. No significant difference between the two groups.	[70]
Watermelon rind	Tensile	Tensile testing at the strain rate of 10 mm/min.	The sample possess an elastic modulus of 1.335 MPa and 0.595 MPa in the dry and wet states, respectively. The stiffness of the scaffold declines upon soaking in water.	[72]
Parsley	Tensile and suture retention	For the tensile tests, wet and dry decellularized parsley stems were cut and attached to the tensile grips 10 mm away from both ends. The tensile tests were performed at a rate of 6 mm/min until failure occurred. Suture retention tests were performed according to BS EN ISO 7198:2017. The tensile tests were performed at a rate of 6 mm/min until failure.	The test results demonstrated that the elastic modulus, UTS, and suture retention strength of the decellularized parsley stems under wet conditions were 5.182 ± 0.856 MPa, 0.471 ± 0.044 MPa, and 0.066 ± 0.029 MPa (0.242 ± 0.067 N), respectively. The percentage elongation of the samples at UTS was 12.3 ± 2.4%. Under dry conditions, the samples were more brittle, giving a significantly higher elastic modulus (32.148 ± 1.649 MPa). The tensile strength of the dry samples was also significantly higher, with a UTS of 1.301 ± 0.051 MPa. The elongation of the dry test samples at UTS was 8.1 ± 0.6%, slightly lower than that observed under wet conditions. The mechanical properties of the developed TEVG were similar to artery-like behavior with a strain of 13% and a UTS of 0.14 MPa.	[74,105]
Spinach	Tensile and suture retention	The samples were attached to the tensile grips from both ends, and the tensile test was performed at a rate of 6 mm/min until failure. For suture retention: wet samples were sutured 2 mm from the top end and attached to the tensile grip. The bottom end of the samples was fixed to the lower grip 10 mm from the end, and suture retention tests were performed at a rate of 0.1 mm/s until the load bearing was reduced to 10% of the maximum force.	The tensile test results showed that the average elastic moduli of the longitudinal and transverse samples were significantly different from each other. The elastic moduli of the longitudinal and transverse testing samples were 0.843 ± 0.096 MPa and 0.250 ± 0.032 MPa, respectively. The UTSs of the longitudinal and transverse test samples were 0.145 ± 0.007 MPa and 0.101 ± 0.006 MPa, respectively. The suture retention strengths of the longitudinal and transverse samples were 0.090 ± 0.017 MPa and 0.073 ± 0.002 MPa, respectively.	[76]
Spinach	Tensile	Samples were stretched in quasi-static mode. All static tests aiming to identify tensile moduli, strength, and ultimate strain were performed with a strain rate of 5%/min and a preload force of 0.01 N.	The tensile modulus of the decellularized scaffolds was 2.2 ± 0.9 MPa, 4-fold lower than that of native primary veins at 10.1 ± 2.4 MPa. Likewise, the decellularized primary vein demonstrated a 3-fold lower ultimate strain and 8-fold lower ultimate tensile strength than the native tissue.	[77]
Celery	Compression	Unconfined compression tests, with a bath chamber. Hydrated samples were evaluated at a displacement rate of 0.01 mm/s up to 50% deformation.	Celery scaffolds after 24 h decellularization had a Young’s Modulus of 46.76 +/− 8.43 kPa in slices cut transversely and 42.51 +/− 7.78 kPa in slices cut longitudinally.	[78]
*Leatherleaf viburnum*	Suture retention	Suture retention tests were performed on plant-based grafts and rat aorta to compare maximum load of 8–0 and 10–0 Prolene sutures. The graft or aorta was clamped to one end of the Testing machine, and the suture holder clamped to the other. Tension was applied to sutures at a rate of 1 mm/s until a tear in the graft or aorta was observed.	Retention force of 8–0 and 10–0 Prolene sutures was 0.74 ± 0.13 and 0.65 ± 0.28 N for plant-based grafts and 1.01 ± 0.17 and 0.32 ± 0.10 N for rat aorta, respectively.	[79]
Pumpkin	Tensile	The mechanical properties of the scaffolds were measured with a strain rate of 5 mm/min.	The coated MgO2-Pumpkin scaffold exhibits higher (~21%) tensile strength (1.71 MPa) compared to the uncoated version (1.42 MPa). The compressive strength of both coated (0.5721 MPa) and uncoated (0.5633 MPa) pumpkin showed no difference.	[82]
Apple	Compression	Custom-built uniaxial compression apparatus, at a constant rate of 3 mm/min.	No significant difference was observed in the modulus between the blank scaffolds (31.6 kPa ± 4.8 kPa) and the cell-seeded scaffolds cultured in non-differentiation medium (24.1 kPa ± 8.8 kPa; *p* = 0.88). In contrast, a significant difference was noted between the modulus of the blank scaffolds (31.6 kPa ± 4.8 kPa) and that of the cell-seeded scaffolds cultured in differentiation medium (192.0 kPa ± 16.6 kPa; *p* < 0.001).	[86]
*Lisianthus* sp.	Tensile	Uniaxial tensile strength at the 1 mm/min rate	Elastic modulus of 31 MPa	[89]
*Bougainvillea* sp.	Tensile	Native and decellularized samples with a load of 500 N and stretching speed of 1 mm min^−1^ at room temperature.	The bracts exhibited tensile strength values (around 1.5 MPa) with no significant difference between native and decellularized.	[90]
Apple	Compression	The coated and uncoated scaffolds were subjected to a strain of 30% at a strain rate of 2 mm/min.	The Young’s modulus for uncoated and coated scaffolds were calculated as 10.13 kPa and 6.78 kPa, respectively.	[91]
*Taraxacum Ruderalia*	Hydrostatic conductivity	The hydrostatic conductivity at the average pressure of lymphatic collectors and density of lymph fluid were measured. The risk of kinking was determined by curving the conduit around a plastic template of predefined decreasing diameter ranging from 50 to 5 mm. As comparative controls for the hydrostatic conductivity and kink resistance test, fresh arteries, veins, and lymphatic collectors were harvested from swine extremities and decellularized using the same method.	The size of the tubes generated ranged between 1 mm and 1 cm, making them candidates for anastomosis of lymphatic collectors, small and large blood vessels. These observations were quantified by the kinking resistance test, where the cellulose tube proved to be statistically equally resistant to kinking as decellularized veins, lymphatic collectors, and arteries. The hydraulic conductivity in the physiologic range of a lymphangion (65 cm H_2_O) and fluid of normal lymph viscosity (0.0018 Pa) was found to be statistically equivalent (α 0.05) to the conductivity of decellularized lymphatic and vascular vessels of the swine.-	[92]
Pomelo	Tensile, shear and compression	The mechanical properties were evaluated using an electromechanical universal tester. For tensile tests, samples were stretched at 2 mm/min until failure. For shear tests, samples were adhered between two pork skin slices and pulled apart at 2 mm/min. For compression tests, samples were compressed at 2 mm/min to 90% strain.	The compressive stress of the decellularized pomelo with MOF and Gel (4.53 ± 0.14 MPa) was much greater than decellularized pomelo (3.59 ± 0.09 MPa) and MOF and Gel (0.28± 0.03 MPa) at 90% strain. The tensile stress of decellularized pomelo with MOF and Gel before fracture could reach 337.52 ± 7.54 kPa, much higher than decellularized pomelo (299.03 ± 10.23 kPa).	[93]
*Leatherleaf viburnum*	Tensile	Samples were subjected to uniaxial tensile testing at a rate of 0.08 mm/s until failure.	Trypsin/Tergitol-, Trypsin/Tergitol/EGTA, and SDS/Tergitol-treated samples exhibited the highest tensile strength and elastic modulus, followed by SDS-treated samples with 6 h of clearing (1.2–2.6 N/mm^2^). Extended clearing times (>12 h) weakened scaffold structure, reducing both tensile strength and elasticity.	[94]
Walnut leaves	Tensile	Native and decellularized samples were evaluated in wet conditions at a displacement rate of 0.1 mm/s and with a preload of 0.1 N.	For native samples, the Young’s modulus was 5.16 ± 0.89 MPa, for the decellularized ones it was 4.17 ± 0.91 MPa. Statistical analyses indicated no significant differences between native and decellularized walnut leaves scaffolds in terms of mechanical properties.	[95]
Water spinach Green onion Water horsetail	Tensile	A tensile test was performed to determine Young’s modulus, tensile strength, and maximum elongation of the plant scaffolds.	Native and decellularized water horsetail showed the highest Young’s module of 343.7 ± 15.6 and 73.9 ± 15.69 MPa respectively, compared to native and decellularized water spinach (21.54 ± 1.18 and 10.35 ± 2.33 MPa) and green onion (19.49 ± 1.38 MPa and 8.42 ± 2.30 MPa) respectively. Water spinach is the most promising graft candidate in suturability tests besides presenting the highest elongation before rupture in tensile test, with a maximum value of 7.31 ± 0.64% after decellularization. In comparison, GO and WH showed similar maximum elongations of 2.80 ± 1.13% and 2.37 ± 0.59%.	[97]

Apple [35,55,66,86,91], *Alstroemeria* sp. [52], bamboo [41], *Borassus flabellifer* [47], carrot [35], and celery [78] were all evaluated in terms of compressive strength, as well as scaffolds of gelatin–methacrylate combined with dill, fennel, and wild fennel [53]. Similarly, compressive properties were also assessed for poly-L-ornithine-laced asparagus [61] and other plant-based scaffolds modified with sodium periodate and APTES or OTS [58] or coupled with chitosan and PRP [57].

In accordance with the intended application, other studies evaluated the tensile strength of their decellularized plant-based matrices, such as *Bougainvillea* sp. [90], cabbage [49], celery [35], chive [45], corn husk [65], *Ficus hispida* [32], jackfruit [65], leatherleaf [94], *Lisianthus* sp. flower stems [89], nopal [70], olive leaves [67], onion [48,97], parsley [74], pomelo [93], pumpkin [82], spinach [31,39,45,54,56,63,76,77], sweet mint [54], tomato leaves [54], walnut leaves [95], and watermelon rind [72].

Some studies also involved the use of cyclic strain tests, as documented for spinach [54], sweet mint [54], stems of *Taraxacum Ruderalia* [92], and tomato leaves [54]. Finally, a suture retention test was specifically reported for leatherleaf scaffolds [79].

In addition to the mechanical properties, the scaffold microstructure also plays a pivotal role for proper cell adhesion and growth. In this regard, SEM microscopy and/or micro-CT have been commonly utilized to analyze the morphology, dimensions, and interconnectivity of the pores compared to the plants’ native vasculature and microporosity. SEM imaging can also be used to observe the cells attached to scaffolds, as a supplementary microscopy technique. AFM microscopy also aids in the topographical characterization of the scaffold and is particularly useful to evaluate the surface roughness. Other common tests of plant-based scaffolds include those that help to characterize them in terms of physical properties, such as water retention, swelling degree (as measurements of porosity and water uptake), degradation rate (to mimic or study the possible changes in mass when the scaffold is placed in biological systems), and contact angle (since hydrophilicity can affect the adhesion of the cells). BET analysis is used to measure the surface area and the porosity of the scaffold via gas absorption, and can be complementary to other techniques to evaluate porosity. TGA is also commonly used to evaluate the thermal stability and decomposition behavior of the scaffold, by measuring weight loss as a function of temperature. Other physical tests aimed at specific applications (mainly vascular grafts) include the burst pressure and leakage tests.

Additionally, chemical characterization of the scaffolds can be performed to analyze the chemical bonds present in the matrix or those created between the native cellulose and the chosen moiety for functionalization. FTIR is used, for example, in cases where there is a chemical change to the cellulosic wall, such as oxidation or functionalization with a chemical group. Similarly, RAMAN spectroscopy can be used along with FTIR, as it gives complementary information. XRD helps to determine the crystalline or amorphous nature of the scaffold and is particularly useful in cases where there is deposition of hydroxyapatite or other crystals. 

As regards scaffolds’ biological properties, a large variety of tests have been reported so far, depending on the intended application, e.g., wound closure analysis, hydrolytic degradation in vivo, angiography, electrical conductivity, antibacterial activity, hemocompatibility, histology, immunohistochemistry, and total protein absorption. The main findings on the biological performance of plant-based scaffolds are specifically addressed and commented on in the following section.

## 4. Discussion

In this scoping review, we identified 71 research articles, published in the last decade, about the use of decellularized plant tissues for mammalian cell reseeding, with potential applications in advanced biotechnological fields, such as tissue engineering, in vitro disease modeling, and the production of laboratory-grown meat. As discussed above, an insight into those 71 articles highlighted a much higher number of plant or vegetable sources and tissues (Table 2): A total of 89 protocols were used for decellularization (with different chemical and/or physical methods), and a multitude of strategies were explored to induce the adhesion of mammalian cells (mostly via surface functionalization) and, as a result, there were high variabilities in both cell responses and degrees of success.

**Figure 6:**
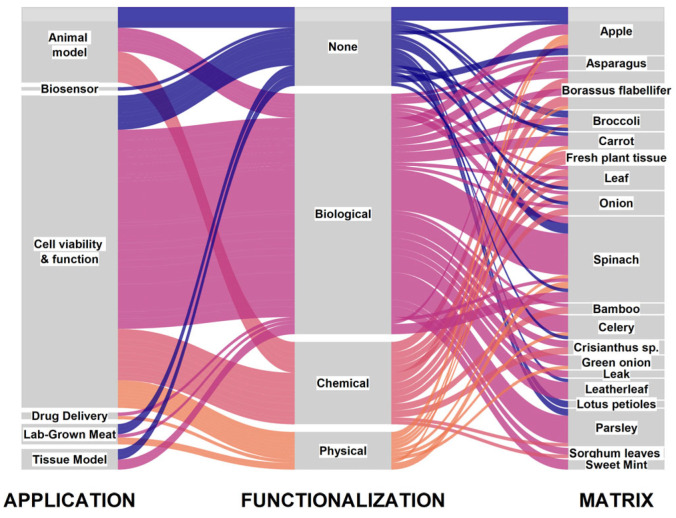
Alluvial diagram highlighting the applications and functionalization methods for commonly used plant matrices.

Therefore, while the technology of plant-based scaffolds for tissue engineering is still in its initial stages, an accurate snapshot of the whole body of the existing literature appears particularly challenging. Figure 6 provides only a partial and simplified overview of the literature, with reference to the most used plant matrices and their functionalization methods, yet we believe it can be useful to stress the dynamicity and attractivity of plant-based scaffolds in biotechnology.

In this review, we gathered basic information on plant sources, decellularization and recellularization methods, and related results, with the aims of highlighting current limitations and opportunities and providing suggestions for future studies. In the following, we discuss in vitro and in vivo results obtained with the use of plant-based scaffolds, relying on either their surface properties or their surface functionalization to promote proper cell interactions. Emerging applications, other than tissue engineering, are also briefly presented.

### 4.1. Cellular Adhesion, Growth, and Differentiation In Vitro

The work of Modulevsky and collaborators was the first showing the potential of plant-based scaffolds for tissue engineering, upon proper surface functionalization. They indeed demonstrated the invasion, proliferation, and viability of human epithelial cells (HeLa) as well as mouse fibroblasts (NIH3T3) and mouse muscle myoblasts (C2C12) on apple scaffolds functionalized with collagen [15]. Other authors then reported the use of both collagen and fibronectin to functionalize spinach leaves, and their results indicated decreased proliferation rates of human prostate cancer cells (PC3) and melanoma cells (SK-MEL-28), probably because of the lower stiffness of the plant scaffolds when compared to rigid substrates [36]. Spinach, sweet mint, parsley, and celery were also functionalized with both collagen and fibronectin to evaluate the cytocompatibility of the supercritical CO_2_ decellularization method, with respect to human dermal fibroblasts (hDFs) [101]. Other authors coupled biological functionalization with physical changes in the matrix, as reported for chitosan–-*Borassus flabellifer* cellulose-derived scaffolds [47]. Fibrin-coated cabbage scaffolds were also exploited for HUVEC proliferation, showing better results compared to control cells cultivated in tissue culture plates (TCPs). Another example comes from the use of chitosan-coated *Alstroemeria* sp. stems for the growth and differentiation of MC3T3, where the chitosan coating rendered the plant-based scaffolds more cytocompatible, increased their mechanical stiffness, and promoted the invasion of the cells into the scaffolds [52].

Some research groups investigated more sophisticated approaches to facilitate cell adhesion, for example, with the use of an RGD–dopamine coating for the attachment of both hDFs and human mesenchymal stem cells (hMSCs) on parsley scaffolds. In the same work, the biomineralization of parsley stems was also found to sustain the attachment of hDFs and hMSCs. Although the biomineralization changed the matrix pore topography and size, this did not negatively affect the cell expansion efficiency, which was higher on the plant scaffolds versus the monolayer culture [30]. The modified RGD–dopamine peptide also showed promising results when applied to *Ficus hispida* scaffolds [32]. In a similar fashion, L-DOPA was applied to spinach and chive scaffolds recellularized with ciPTEC, but in this case, the results were not as promising as expected [45]. Another polyaminoacid exploited for cell adhesion and growth is poly-L-ornithine, which was applied to asparagus stems for the culture of rH-NSC [61]. An additional surface functionalization strategy involved the use of polyaniline and GO to enhance the adhesion of Schwann cells to *Lisianthus* stems [89], which were thus found to be promising candidates for fabricating multichannel scaffolds for neural tissue engineering.

The importance of surface functionalization was further highlighted in several research studies directed to induce cell differentiation. For instance, apple scaffolds were shown to sustain the osteoblastic differentiation of hiPSCs [33], while poly-L-lysine-functionalized apple scaffolds were adopted for the adipogenic differentiation of 3T3-L1 cells. The same functionalization moiety was also applied to carrot scaffolds seeded with MC3T3-L1 cells and celery scaffolds seeded with L929 cells to study osteogenic and [56] chondrogenic differentiations, respectively [35]. Another approach included the use of grape seed extract on a date palm matrix for the differentiation of MG63 cells [62], while watermelon rind functionalized with polydopamine was tested with hMSCs. An alternative chemical oxidative functionalization treatment was also reported to aid in the osteogenic differentiation of rASCs [41].

However, numerous studies also demonstrated how the given structural features of the plant matrix, such as the surface roughness, porosity, and mechanical properties, may suffice to induce and sustain cell growth and differentiation, without the need of further functionalization. As an example, uncoated spinach scaffolds were found to induce both the mineralization and osteogenic differentiation of hBM-MSCs [39]. Similarly, uncoated cabbage leaves showed promising results as scaffolds for osteogenic differentiation, as mesenchymal stem cells grown on the scaffolds demonstrated higher calcium mineralization and ALP activity, as well as higher expression of bone-related genes, compared to those of cells on TCP [49]. This was likely related to the peculiar roughness and porosity of the cabbage matrices. Similarly, the use of onion skin promoted a greater differentiation of hBM-MSCs compared to that of the TCP control [48]. In this context, apple-derived scaffolds were also physically stimulated through cyclic hydrostatic pressure, as a way to promote cell replication, ALP activity, and mineralization over time [55]. In other studies, human dental pulp stem cells (hDP-SCs) were cultured on nopal, *Beaucarnea recurvata*, and spinach scaffolds. In all three cases, the decellularized plant scaffolds were able to sustain the attachment and growth of the cells. Regarding the nopal scaffold, the decellularization procedure and the matrix by itself did not induce any inflammatory response [70]. As for *Beaucarnea recurvata*, the leaves were functionalized with grape seed proanthocyanidin extract, which enhanced the odontoblastic and odontogenic transformations of the hDP-SCs [80], while cells grown on the 5% HCl-treated spinach scaffold [88] showed significantly increased osteogenic differentiation compared to that of the TCP control, as evaluated via alizarin red and osteonectin expression. In a recent comparative study between celery and gelatin–methacrylate (GelMA) scaffolds, the authors focused on the role of mechanical cues in cell differentiation, highlighting that the stiffness, itself, of the plant-based scaffolds could favor the osteogenic differentiation of hASCs, while cells on the GelMA scaffolds underwent a chondrogenic differentiation pathway [78].

When analyzing the reported cell studies about plant-based scaffolds, it is worth observing that several groups tried to take advantage of the anisotropy and orientation of plant tissues to induce cell alignment. For example, the use of RGD-DOPA functionalization on a *Solestenemon scutellarioides* matrix allowed the observation that hDF cells predominantly grew close to plant’s stomata or within grooves [30]. Fibronectin-coated green onion scaffolds demonstrated the alignment of C2C12 and human skeletal muscle cells (hSMCs) [34], while the use of lotus petioles, coated with either ECM molecules or GO, promoted the neural alignment of PC12 cells [68]. In another work, the authors used sorghum leaves mixed with a poly (PEGMEMA-*r*-VDM-*r*-GMA) copolymer and the RGD peptide to promote the adhesion of hESCs and their differentiation into oriented muscle cells; the cells showed early myogenic differentiation and contraction. The effects of plants’ natural grooves and valleys on the cell behavior were also explored for different algae, where the successful re-seeding of hDFs was found to be dependent on the native morphology of each type of algal scaffold [73]. Finally, transglutaminase-crosslinked gelatin-coated parsley scaffolds could profit from the biological adherence motifs granted by the gelatin and the natural architectural organization of the parsley for the growth, differentiation, and orientation of C2C12 cells [75].

The potential of plant-based scaffolds for mammalian cell culture was also addressed in studies about cell contraction. A fibronectin-coated spinach scaffold could permit the contraction of human pluripotent stem cell-derived cardiomyocytes (hPS-CMs), with a peak at 10 days of culture until day 17 [31]. In another work, a spinach matrix functionalized with either fibronectin or collagen or without any coating was used to study the contraction of human-induced pluripotent stem-cell-derived cardiomyocytes (hiPS-CMs), highlighting no difference in cell contraction on day 7 and day 21 [38].

As is widely known, proper vascularization is pivotal to the success of any tissue-engineering approach. In this regard, borrowing the native vasculature of plant tissues to enhance the regeneration of tissues and organs has been the subject of several studies. For instance, the inner surface of spinach scaffolds was coated with fibronectin, seeded with human umbilical vein epithelial cells (HUVECs), and evaluated in perfusion experiments to evaluate the feasibility to mimic the mammalian vascular system. A modified *Aptenia cordofilia* leaf was also recellularized with MDA-MB321, with the same aim. However, this work showed no difference in morphology or cell density between the cells grown on the scaffolds and those grown on TCP [40]. Other authors exploited plant matrices as potential scaffolds to produce engineered vascular grafts. Leatherleaf tubes functionalized with glutaraldehyde-crosslinked gelatin and rEC showed suitable tensile and rupture properties as small-caliber vascular grafts [63]. Additionally, decellularized parsley seeded with HUVEC and L929 cells was utilized to form a vascular graft. In this case, the cells were able to secrete their own ECM onto the scaffold without the need of any functionalization [74]. In a similar fashion, leatherleaf tubes coated with fibronectin and gelatin and recellularized with rAEC and rVSMC showed promising results both in biological and mechanical tests [79]. The same matrix type showed ideal mechanical behavior in suture retention tests, indicating it as a good candidate for the development of vascular grafts [94]. A recent comparative report about spinach, green onion, and water horsetail scaffolds indicated that the water horsetail one was the least promising in relation to mechanical properties, while both the spinach and green onion scaffolds presented more cells and higher proliferative proteins compared to those of the control group [97].

Interestingly, the peculiar vasculature of plant tissues was also investigated for kidney tubule engineering. However, obtained findings for spinach and chive matrices proved that the vasculature of these plants is not ideal to replicate the structure of kidney tubules [45]. Conversely, the recent development of potential grafts for lymphatic drainage from Poly-D-lysine-treated *Taraxacum Ruderalia* stems suggested a new application in the field of lymphedema treatment [92].

### 4.2. In Vivo Results

While the in vitro potential of plant-based scaffolds for tissue engineering has been widely explored, few research groups have investigated the tissue responses to those scaffolds upon implantation in animal models.

In this regard, promising data were first reported for leucocytes, such as neutrophils and eosinophils, which were shown to invade an uncoated apple scaffold after 1 week of subdermal implantation in mice, with the immune response by the 8th week being under control and the tissue appearing as normal [29]. Further studies on apple-derived scaffolds seeded with MC3T3-E1 preosteoblasts were also performed to assess their osteogenic potential in vivo after 8 weeks of implantation [86]. In the context of non-load-bearing bone tissues, another study demonstrated the survival of hiPSC-derived osteoblasts on scaffolds derived from various plants, upon implantation in a rat calvarial defect model [33]. In a similar context, the in vivo degradation of oxidized-bamboo scaffolds was also evaluated after 4 weeks of subcutaneous implantation, showing the presence of macrophages in both the interior and at the periphery of the scaffolds [41]. While in vitro results of oxidized-APTES scaffolds from *Borassus flabellifer* proved osteoblast invasion, proliferation, and differentiation even higher than those of oxidized-OTS ones, a minimal inflammatory response was elicited by both scaffolds when implanted in a rat model [58].

Notably, a recent study showed that liver function could improve in mice with acute liver injury after the transplantation of apple scaffolds loaded with rASCs, which was accompanied by a downregulation of liver inflammation [83].

Pumpkin scaffolds functionalized with magnesium oxide nanoparticles led to promising in vitro results and were then transplanted in mice, where they were found to support fibroblast migration, collagen deposition, and accelerated wound healing [82]. Similarly, in vitro results about the hemocompatibility and cytocompatibility of *Ficus benjamina* scaffolds were later confirmed after subdermal implantation in rabbits. In vivo tests also showed the invasion of neutrophils into the scaffolds after 20 days [85].

### 4.3. Other Emerging Biotechnological Applications

A prospective use of plant-based scaffolds lies in the development of laboratory-grown meat (also called cellular agriculture), as demonstrated by some recent results on matrices such as spinach [46,64], broccoli [59], corn husk [65], and jackfruit rind [65] grown with bovine PB-SCs, as well as on chicken-scaffolded celery [81]. As a proof of concept, other potential matrices for meat cultivation include shiitake, oyster, and wood ear mushrooms explored for the growth of C2C12 cells [98] and natural leaf veins for the growth of PC-SCs and dASCs [87]. Similarly, other tests regarding not only the cytotoxicity of the scaffolds but also their degradability under gastric conditions indicated that gelatin- and alginate-coated apple scaffolds could be used for this purpose [91].

A further emerging area of application regards the development of in vitro tissue models of both diseased and healthy tissues for basic research and/or drug testing. Spinach leaves were particularly investigated in this context. As an example, spinach-based scaffolds were developed to study the behaviors of PC3 prostate cancer cells and SK-MEL-28 melanoma cells and their reactions to radiation and chemotherapy [36]. Another cancer tissue modeled on spinach-based scaffolds was lung tissue, with A549 and BEAS-2B cells [84]. Spinach leaves coated with fibronectin and seeded with adenocarcinoma epithelial cells were also found to allow cells to align along the direction of the mechanical stretch, thus behaving in a physiologically relevant manner for lung tissue [54]. Continuing with spinach-based matrices, the co-culture of breast cancer spheroids and the plant vasculature under flow resulted in a closer-to-reality growth model [77]. Finally, spinach scaffolds were also used as a model to study amyloid deposits, although this approach met some setbacks in image collection [96].

With regard to other plant-based scaffolds used for disease modeling, hairy tomato leaves treated with plasma to increase hydrophilicity and then recellularized with HepG2 were recently utilized to mimic human hepatocarcinoma for drug-screening applications [60]. Further plasticity was shown by plasma-modified eggplant leaves, which appeared to be a promising platform to study liver cancer cells and their responses to prilocaine [71].

As for the development of healthy tissue models, fennel and dill leaves were used to create a vascular network in a methacrylate–gelatin hydrogel and recellularized with 3T3-L1 to obtain a model for adipose tissue [53]. Nanoamyloid- and nanohydroxyapatite-coated celery recellularized with MC3T3, HUVEC, C2C12, and rBMSC cells also showed that nanoamyloids by themselves were effective in promoting cell adhesion, growth, and differentiation. This finding, coupled with results regarding osteogenesis in vitro and in vivo, indicated the widespread applicability of this particular coating for modeling non-load-bearing bone tissue [56].

The design of drug delivery platforms represents an additional area where plant-based scaffolds could find applications. In one study, onion leaf and skin were loaded with PLGA-based rapamycin nanoparticles and implanted into the rat infrarenal inferior vena cava (IVC). After 14 days, it was shown that these matrices could be used as vascular patches in rat venoplasty and that their surfaces could be modified for drug delivery [42]. Likewise, in another work, the use of *Epipremnum aureum* leaves laced with IL-33 was proposed as a model to reduce venous neointimal hyperplasia [51]. While apple-derived scaffolds were investigated for gastrointestinal probiotic delivery [66], other authors modified plant stems with chitosan and then loaded them with PRP, suggesting their potential use as protein- or drug-releasing platforms [57]. Spinach-based matrices loaded with ascorbic acid were also found to enhance the growth, proliferation, and collagen production of L929 fibroblasts [76]. In another work, pomelo peels were first functionalized with PVA-TSPBA hydrogel and then loaded with antibacterial gallic acid/copper MOFs [93]. A parallel line of research regarded the usage of plant-based scaffolds as wound-healing patches, such as the ones obtained from walnut leaves [95].

Lastly, a novel attractive biotechnological application of plant-based scaffolds concerns the design and development of biosensors. In this context, broccoli was recently used for enzyme immobilization for the sensing of glucose for cell culture viability and was tested with NHDF and human Lin Sca-1pos cardiac mesenchymal cells (cMSCs) [43].

### 4.4. Limitations

This scoping review is not exempt of limitations. Due to the increasing interest in plant-based scaffolds for tissue engineering and related biotechnological applications, we might have missed a relevant number of publications. While we were unable to include publications published after April 2025, we also decided to exclude full-text articles that were not accessible by our institution. Notwithstanding, according to the references cited in the identified articles, we believe that our article selection was accurate enough to map the research conducted in this area and identify current limitations and gaps in knowledge, which were the main aims of this review.

Starting from the matrix selection, the literature shows a large variety of plants and vegetables, as well as fungi and algae, experimented on so far. While research efforts on different plants are needed to identify the most successful matrices for given applications, we believe that a more careful choice of plant sources, in terms of species, plant tissues, geographical locations, and cultivation techniques, would be desirable to reduce the high variability in the findings. Moreover, future economic and environmental impacts associated with the production of plant-based scaffolds should be considered. Many studies indeed used plants and vegetables that have a commercial value (e.g., in the food industry), which raises concerns about potential costs and resource competition. As research in the field of plant-based scaffolds advances, it will be essential to prioritize strategies that utilize plant parts regarded as waste or that have a lower economic value to ensure the scalability of the technology, without causing disruptions to existing agricultural and commercial fields.

In addition, our review pointed out that a significant limitation of plant-based scaffolds also lies in the complete lack of standardization for the decellularization, functionalization, and recellularization of matrices, which may lead to inconsistent results among different studies, even if dealing with the same type of plant tissue and the same intended application. This highlights the need to establish standardized, uniform procedures, also by potentially focusing on specific source materials, to improve the reproducibility and facilitate a broader adoption of plant-based scaffolds.

Finally, in vivo responses to plant-based scaffolds have not been widely addressed. Thus, further exploration is needed for a deeper understanding of the actual potential of plant-based matrices for clinical translation.

## 5. Conclusions

This review aimed to provide a potential tool for researchers that want to approach and explore the possibility of using plant-based matrices for tissue engineering and other advanced biotechnological applications. Currently, the large diversity in plant matrices, protocols, and testing reported in the literature makes it difficult to reach consensus and definitive answers on the performance of plant-based scaffolds in selected contexts. While the use of plant-based scaffolds for the engineering of load-bearing tissues remains a challenge, the overall biotechnological potential of plant-based scaffolds is undeniable, as demonstrated by the research findings (in vitro and in vivo) herein gathered and discussed. Additionally, the use of plant-based scaffolds could also enter and morph clinical practice via several applications here analyzed, such as tissue regeneration, tissue and disease modeling, drug delivery, and one that we did not discuss herein, i.e., the ex vivo proliferation of cells before implantation or treatment. We believe that the selection of plant sources with low economic and environmental burdens, the standardization of the involved methods (i.e., for decellularization, functionalization, and recellularization), and the conduction of further in vivo analyses are prerequisites that should be pursued by future investigations. Widespread application of these techniques is far from reality as of now. However, given the economic and accessibility challenges that impact any research at any time, we encourage new researchers to look at such technologies that can potentially reshape the clinical landscape, regardless of the difficulties they may face along the way.

## Figures and Tables

**Table 1:** Search strategies and handles by database.

Database	Search Handle	Used Website Filters
PubMed	<< (((decellularized) AND (scaffold)) AND ((plant) OR (vegetable))) >>	None
Science Direct	<< decellularized AND scaffold AND (plant OR vegetable)>>	Article type > Research article
Scopus	<<(TITLE-ABS-KEY (decellularized) AND TITLE-ABS-KEY (scaffold) AND TITLE-ABS-KEY (plant) OR TITLE-ABS-KEY (vegetable) AND NOT TITLE-ABS-KEY (review))>>	None
Sage Journals	<<‘plant’ OR ‘vegetable’ AND ‘decellularized’ AND ‘scaffold’>>	Article type > Research article

**Table 2:** Main characteristics of the sources of evidence: first author (last name) and year of publication, plant matrix, decellularization protocol, cell line(s) or animal model, cell interactions and main results.

Author, Publication Year	Plant Matrix	Decellularization Protocol	Cell Line(s) Used/ Animal Model	Cell Interactions/ In Vivo Study	Summary of Findings	Ref.
Modulevsky, 2014	Apple	Chemical: SDS	NIH3T3, C2C12, and HeLa	Cellular adhesion, proliferation, and invasion	Apple scaffolds were functionalized with either Type I collagen, glutaraldehyde +NaBH4, or control (PBS). After 4 weeks of culture, all three cell lines showed similar proliferation in the biological and chemical-modified scaffolds. Particularly, NIH3T3 and HeLa cells also proliferated on the unmodified scaffolds.	[15]
Modulevsky, 2016	Apple	Chemical: SDS > PBS	Wild-type and C57BL/10ScSnJ mice	Subcutaneous implantation	Scaffold biocompatibility and cell infiltration were examined with H&E staining of fixed cellulose scaffolds following their implantation. The results demonstrate that by 8 weeks post implantation, the host accepted the cellulose scaffold.	[29]
Fontana, 2017	Bamboo, Parsley, Vanilla, *Anthurium magnificum*, *Anthurium waroquaenum*, *Calathea zebrina*, *Laelia ancepts*, *Schoenoplectus tabernaemontani*, and *Solenostemon scutellarioides*	Chemical: SDS > Triton X-100 + 10% Sodium Hypochlorite > n-Hexane	hDF and MSC	Adhesion	Decellularized plants coated with the RGD–dopamine conjugate (RGDOPA) supported the adhesion of hDF and MSCs, while noncoated plants did not support cell attachment on parsley stems. Decellularized plants were also functionalized via biomineralization; these plants also supported the attachment of hDF.	[30]
Gershlak, 2017	Spinach, Parsley, *Artemisia annua*, and Peanut Hairy Leaves	Chemical: [n-Hexane > PBS] > Triton X-100 + Sodium hypochlorite	HUVEC and hPS-CM	Cytocompatibility, adhesion, proliferation, and contraction	HUVECs were seeded on the inside to mimic the endothelium and hPS-Cm on the outside. After 3 days, the hPS-CM formed clusters; after 5 days, they spontaneously contracted. There was no difference between clusters grown on the decellularized scaffolds and on TCP.	[31]
Adamski, 2018	*Ficus hispida*, *Paquira aquatica*, and *Garcinia* sp.	Chemical: (a) SDS > Nonionic surfactant + Sodium hypochlorite > TRIS-HCl [10 mM, pH 8.5] (b) Sodium hypochlorite + Sodium Bicarbonate + Temperature	hDF and MSC	Cell viability and cell adhesion	The hDFs had higher metabolic activities on bleached scaffolds than on SDS-treated ones. RGD–Dopamine-coated *F. hispida* leaves showed the adhesion and growth of MSCs.	[32]
Lee, 2019	Apple, Broccoli, Sweet Pepper, Carrot, Jujube, and Persimmon	Chemical: SDS > Ethanol	hiPSC Sprague–Dawley rats	Cell attachment and proliferation, osteogenic differentiation. Rat calvarial defects	Apple, carrot, and persimmon scaffolds were evaluated for cell viability, all with satisfactory results. Apple scaffolds were used for osteogenic differentiation. Elevated levels of mRNA expressed in a time-dependent manner were recorded by osteogenic markers, such as osteocalcin (OCN), and type I collagen (COL-1) was constant.	[33]
Cheng, 2020	Carrot, Broccoli, Cucumber, Potato, Apple, Asparagus, Green Onion, Leek, and Celery	Chemical: SDS	C2C12 and hSMC	Cell attachment, proliferation, and alignment	The outer white section of the green onion had a microstructure that guided C2C12 cell differentiation into aligned myotubes. Quantitative analysis of both cell lines’ alignments revealed an almost complete anisotropic organization compared to that of 2D isotropic controls.	[34]
Contessi-Negrini, 2020	Apple, Carrot, and Celery	Chemical: SDS > CaCl_2_	3T3-L1 preadipocytes, MC3T3-E1 pre-osteoblasts, and L929 fibroblasts	Adhesion, proliferation, and differentiation (adipogenic, osteogenic, and guided anisotropy)	Polylysine-coated samples. No scaffold showed cytotoxic effects. In terms of adipogenic differentiation, higher metabolic activity values were detected in TCP wells compared to the apple scaffolds. There was no difference between the MC3T3-E1 cells grown on the carrot scaffolds and the ones on TCP. For the L929 cells and celery, there was a higher metabolic activity in TCP wells, but the percentage increase in metabolic activity at t = 14 days compared to that at t = 1 day was significantly higher on celery-derived scaffolds. Celery scaffolds also offered the possibility to align the cells.	[35]
Lacombe, 2020	Spinach, *Solanum lycopersicum*, *Echinodorus grisebachii*), *A. Borealis*, and Luckly Bamboo	Chemical: n-Hexanes > SDS > Triton X-100 + Sodium hypochlorite	SK-MEL-28 PC3	Tissue/disease model	The mechano-regulation of both cell lines on decellularized spinach-leaf scaffolds was decreased compared to cells deposited on standard rigid cell culture substrates.	[36]
Phan, 2020	Tobacco BY-2 and Rice (Cells) and Tobacco Hairy Roots (Tissue)	Physical + Enzymatic: (Lyophilization > DNase)	hFF and THP-1	Cellular attachment and macrophage response test	When exposed to decellularized BY-2 cell-derived matrices, monolayer cultures of hFFs maintained or increased metabolic activity. Furthermore, hFFs were able to attach, spread, and proliferate when cultured with the decellularized BY-2 cell-derived matrices in the aggregate mode. Directly treating THP-1-derived macrophages with the BY-2 cell-derived matrices for 48 h resulted in increased TNF-α secretion as compared to that in the untreated control group, indicating the possible presence of endotoxin remnants from the decellularization.	[37]
Robbins, 2020	Spinach	Chemical: [n-Hexane > PBS] > SDS > Triton X-100 + Sodium hypochlorite > TRIS Buffer	hiPS-CM	Cell viability, attachment, proliferation, and contraction	Similar cell viabilities between the fibronectin-coated, collagen IV, and uncoated-spinach decellularized scaffolds. No differences in contraction were found between coated leaves, (TCPs), noncoated leaves, or noncoated TCP at most points (on all but day 14).	[38]
Salehi, 2020	Spinach	Chemical: n-Hexane > SDS > Triton X-100 + Sodium hypochlorite > Hexane > Sodium hypochlorite	BM-MSC	Biocompatibility, osteoinductivity, and osteogenic differentiation	The staining results showed that the cells spread on the surface of the scaffold and did not aggregate. Additionally, ALP activity and calcium content measurements in BM-MSCs cultured on the spinach leaf decellularized scaffold on day 18 were significantly higher than those of BM-MSCs cluttered on the TCP. The expression levels of Runx2, osteocalcin, and Col-I genes in the BM-MSCs cultured on the decellularized scaffold at days 9 and 18 were significantly higher than those of the BM-MSCs cultured on the TCP.	[39]
Wang, 2020	*Aptenia cordifolia*	Chemical: n-Hexane > SDS > TritonX-100 + Sodium hypochlorite	MDA-MB23-expressing GFP	Graft tissue	Specially grown and modified *Aptenia cordifolia* plants were decellularized to create a grafted scaffold with double-ended vascularity. Characteristic spindle-shaped green fluorescent cells were readily observed on both the TCP and on the decellularized grafted scaffolds.	[40]
Aswathy, 2021	*Bambusa vulgaris*	Chemical: (a) SDS + Sodium hypochlorite (b) Triton X-100 + Sodium hypochlorite (c) SDS + Triton X-100 (d) Sodium hypochlorite	rA-MSC Wistar rats	Cytocompatibility, cell viability, and osteogenic differentiation Subcutaneous implantation	Oxidized bamboo scaffolds had better MSC adhesion, viability, and osteogenic differentiation than non-oxidized ones. The animal test showed the scaffolds were able to induce angiogenesis and were biocompatible and biodegradable.	[41]
Bai, 2021	Onion Leaf and Onion Skin	Chemical: SDS > PBS > Sodium hypochlorite	Male Sprague–Dawley rat	Inferior vena cava patch venoplasty model	The onion leaf was decellularized, and the scaffold was loaded with polylactic-co-glycolic acid (PLGA)-based rapamycin nanoparticles. Both leaf- and onion-cellulose-laced scaffolds showed decreased neointimal thickness, with the leaf scaffolds also showing fewer CD68+ cells and PCNA+ cells.	[42]
Cancelliere, 2021	Broccoli	Chemical: SDS	NHDF human Lin^−^ Sca-1^pos^ cardiac mesenchymal cells (cMSC)	Biosensor and in vitro toxicity model of cellular attachment and growth	Microstructured scaffolds from stalks of broccoli, named BrcS, were either functionalized for the production of enzymatic 3D biosensors to monitor glucose uptake over time or preconditioned to be used as 3D scaffolds for cMSC cultures. After the preconditioning of the broccoli scaffold with the cell culture medium, it was able to support the cell attachment and growth of cMSCs.	[43]
Harris, 2021	Spinach, Sweet Mints, Celery, and Parsley	Physical vs. Chemical: (a) ssCO_2_ (b) SDS	hDF	Cell viability, attachment, and proliferation	The hDFs were seeded on and attached to scCO_2_-decellularized scaffolds, showing viable cells at 14 days and able to respond to a drug stimulus. As per the authors’ recollection, further analyses are needed to evaluate the influence of the ssCO_2_ decellularization on seeded cells.	[44]
Jansen, 2021	Chive and Spinach	Chemical: n-Hexane > SDS	Tetramethylrhodamine-isothiocyanate (TRITC)-labeled (ciPTEC)	Differentiation into possible vascular grafts	L-Dopa coated samples allowed renal cells to grow on the lumens of chive and spinach petioles, but they did not reach the spinach leaf vasculature through petiole injection. Although recellularization was performed successfully, both spinach and chive tissues quickly disintegrated in the culture. Unsuitable for kidney tubule tissue engineering.	[45]
Jones, 2021	Spinach	Chemical: n-Hexane > SDS > Triton X-100 + Sodium hypochlorite	BP-SC	Lab-grown meat	Cells grown on the decellularized scaffolds showed comparable cell viability compared to cells grown on TCP. Moreover, there was evidence of increased cell differentiation over time.	[46]
Mahendiran, 2021	*Borassus flabellifer*	Chemical: SDS	L929	Cytocompatibility, cell viability, and attachment	Significant increase in cell population on day 5 on the chitosan-decellularized samples compared to the uncoated scaffolds.	[47]
Salehi, 2021	Onion	Chemical: n-Hexane > SDS > Triton X-100 + Sodium hypochlorite > Hexane	BM-MSC	Osteogenic differentiation	ALP activity, calcium deposition, and expressions of bone-related genes, such as *Runx2*, *ALP*, osteocalcin, and Collagen type-I (*col-I*), were higher on onion-decellularized scaffolds than on TCP.	[48]
Salehi, 2021	Cabbage	Chemical: n-Hexane > PBS > SDS > Triton X-100 + Sodium hypochlorite > H_2_O > Hexane	BM-MSC	Osteogenic differentiation	BM-MSCs’ ALP activity and mineralization were increased significantly for cells cultured on the decellularized cabbage leaves compared to those of the cells cultured on TCP. The same applied to the genes: Runx2, *ALP*, collagen-1 (*Col-I*), and osteocalcin.	[49]
Walawalkar, 2021	*Brassica oleracea*	Chemical: SDS > Triton X-100	HUVECs	Cell attachment and proliferation	The fibrin-coated cabbage leaf scaffold showed no cytotoxicity and was able to maintain the metabolic activities and identity.	[50]
Xie, 2021	*Epipremnum aureum*	None	A human spiral saphenous vein graft (SVG) implanted in the popliteal vein was harvested from a patient with trauma. Male Sprague–Dawley rats	Drug delivery of IL-33	Plant leaves absorbed with rhodamine, distilled water (control), rapamycin, IL-33, and IL-33 antibodies were implanted into the rat IVC. There was a large number of IL-33 (*p* = 0.006) and IL-1β (*p* = 0.012) positive cells in the human SVG neointima compared to those in the human great saphenous vein.	[51]
Esmaeili, 2022	*Alstroemeria* Flower	Chemical + Physical: SDS > NaOCl + NaOH at 60 °C	MC3T3	Cytotoxicity, cell attachment, and differentiation	Both chitosan-coated and uncoated scaffolds were cytocompatible. Chitosan-coated plant-based scaffolds had increase roughness, potential swelling, degradation, diffusion, mechanical behavior, and a porous structure when compared to uncoated scaffolds. Chitosan-coated samples also showed good attachment, proliferation, and migration.	[52]
Grilli, 2022	Fennel, Wild Fennel, and Dill	Chemical: SDS + CaCl_2_	3T3-L1 preadipocyte murine cell	Direct cytocompatibility and adipogenic differentiation	No significant differences in cytocompatibility among the three matrices. Adipogenic differentiation studied on dill samples showed greater metabolic activity.	[53]
Harris, 2022	Spinach, Sweet Mint, and Tomato Leaves	Chemical: n-Hexane > SDS > Sodium hypochlorite	A549	Disease model/cancer model	A549 lung cells seeded on stretched scaffolds displayed modified cellular morphologies, like that of cells under strain constraints. Custom-built machine could help to mimic breathing motions. Also, cells seeded on the scaffold could sense the mechanical strain, as demonstrated by a nuclear reorientation perpendicular to the strain direction, a nuclear location of YAP, and increased expression of YAP target genes, a high cytoplasmic calcium level, and elevated expression levels of collagen genes (COL1A1, COL3A1, COL4A1, and COL6A), with increased collagen secretion.	[54]
Leblanc-Latour, 2022	Apple	Chemical: SDS > CaCl_2_ > Ethanol	MC3T3-E1 Subclone 4	Osteogenic differentiation	The application of hydrostatic pressure significantly increased the density of cells after 1 week compared to static condition. Same for ALP activity, Alizarin Red.	[55]
Li, 2022	Celery	Chemical + Enzymatic: (SDS > Triton X-100 + Sodium hypochlorite > Lysozyme > CaCl_2_)	MC3T3-E1, HUVEC, and C2C12 rBM-MSC Mouse RBC Male rats	Biocompatibility, orientation, and arrangement study on RBC adhesion, and osteogenesis in vitro. Evaluation of osteogenesis in vivo	Since day 1, both nanoamyloid-loaded scaffolds and nanohydroxyapatite loaded scaffolds showed greater live cell numbers than decellularized celery, but with no difference among the former two groups. HUVEC and C2C12 myoblasts on celery scaffolds can grow and form a two-layer tube-like structure along plant stems and a membrane-like structure on leaves. Nanohydroxyapatite crystals deposited on amyloid further prompted the osteogenic differentiation of preosteoblasts. Adhesion of rBM-MSC was increased by the presence of nanoamyloids. Compared to decellularized tissue, there were greater expressions of *Osx*, *Alpl*, and *Runx-2* in MC3T3E1 cells seeded on scaffolds with nanoamyloids and nanohydroxyapatite. In vivo experiments proved successful trabeculae regeneration on the scaffold, with nanohydroxyapatite scaffolds showing infiltration of ingrown cells and the newly formed collagen matrix.	[56]
Mahendiran, 2022	*Borassus flabellifer*	Chemical: SDS	MG63 Wistar rats	Cytotoxicity, cell viability, and biocompatibility in vitro and in vivo (subcutaneous implantation). Drug delivery of growth factors from PRP	All the scaffolds (Cellulose, Cellulose–PRP, Cellulose–Chitosan, and Cellulose–Chitosan–PRP) showed no evidence of cytotoxicity. The addition of PRP proved to aid cell viability. The best performance in terms of cell viability was the from the Cellulose–Chitosan–PRP scaffold, followed by the cellulose–PRP, the cellulose–chitosan, and, finally, the cellulose one. The scaffold groups Cellulose–PRP and Cellulose–Chitosan–PRP were able to release PDGF-BB.	[57]
Mahendiran, 2022	*Borassus flabellifer*	Chemical: SDS	hRBC MG63 Raw 264.7 Wistar rats	Hemolytic assay Osteogenic differentiation In vitro inflammatory response In vivo studies	All three scaffolds (oxidized, oxidized + APTEs, and oxidized + OTS) showed to be non-hemolytic and hemocompatible. The oxidized, APTES, and OTS-treated scaffolds showed good cellular adhesion, proliferation, and differentiation of osteoblasts. Animal models demonstrated angiogenesis, degradation, and compatibility with native collagen matrix.	[58]
Thyden, 2022	Broccoli	Chemical: SDS + Tween-20 + Sodium hypochlorite	PB-SC	Lab-grown meat	Cell adhesion was observed, and cell death was limited to 2.55 ± 1.09%. An average cell death of 2.55 +/− 1.09% was observed across all the replicates. There was no significant difference between the different animals of origin. There was an average of 2818 +/− 1062 cells/mm^2^ for all the samples.	[59]
Ahmadian, 2023	Tomato Leaves	Chemical: SDS > Sodium hypochlorite > n-Hexane	hFF HepG2	Cytotoxicity and cell viability. Tissue/Disease model	Over a 7-day culture, the viability of hFF cells seeded on the decellularized tomato leaves increased. Moreover, HepG2 cells formed colonies on both the decellularized scaffolds and the TCP controls. Vitality staining showed that even if HepG2 cells grew faster on TCP, there were also more dead cells when compared to the decellularized scaffold. HepG2 seeded on these scaffolds improved the cells’ response to the drug and increased cell survival in comparison to TCP.	[60]
Couvrette, 2023	Asparagus	Chemical: SDS > CaCl_2_ > Ethanol	Adult rat hippocampal neural stem cells	Attachment, proliferation, and differentiation	NSCs differentiated on the poly-L-ornithine-coated asparagus scaffold showed significant increases in their expressions of neuron-specific beta-III tubulin and glial fibrillary acidic protein compared to TCP, indicating that the scaffold may enhance the differentiation of NSCs toward astrocytic and neuronal lineages.	[61]
Galefi, 2023	*Phoenix dactyliferous*	Chemical: SDS	MG63	Cell viability and cell adhesion, osteogenic differentiation	The cells were evaluated on decellularized date scaffolds and decellularized date scaffolds treated with grape seed proanthocyanin extract. Cells cultivated on the later retained a differentiated phenotype, as evidenced by the presence of pseudopodia; moreover, the cell density on this scaffold was greater than that on the non-treated ones. In terms of osteogenic differentiation, there was a greater deposition of calcium on the scaffolds treated at days 7 and 14 of culturing than the non-treated ones, yet this difference disappeared after 21 days of culturing. Higher ALP activity was found on the treated scaffolds. The same applies to the expressions of Col1A and OCN genes.	[62]
Gorbenko, 2023	Leatherleaf, Spinach, and Parsley	Chemical: (a) SDS > clearing solution (b) SDS > EGTA > clearing solution (c) Tergitol > EGTA > clearing solution (d) Tergitol > SDS > clearing solution	rEC	Adhesion, proliferation, and alignment.	Decellularized leatherleaf, spinach, and parsley were wrapped once around an acrylic rod to form a 3D straw-like structure and then coated with fibronectin and cross-linked gelatin. The rate of proliferation was lower on leatherleaf 2D scaffolds when compared with ECs cultured on TCP. ECs were successfully seeded on 3D grafts made from SDS- and SDS/EGTA-decellularized leatherleaf	[63]
Jones, 2023	Spinach	Chemical: (a) n-Hexane > SDS > Tx100 + Sodium hypochlorite (b) n-Hexane > SDS > PS20 + Sodium hypochlorite (c) PS20 + Sodium hypochlorite	PB-SC	Cultured meat	The cells seeded on non-decellularized scaffolds did not attach, whereas as the cells seeded on the decellularized scaffolds did adhere. The implementation of PS20 as a secondary decellularization agent did not appear to affect cell viability.	[64]
Perreault, 2023	Corn Husk and Jackfruit Rind	Chemical: SDS + Polysorbate-20 + Sodium hypochlorite	PB-SC QM7	Cultured meat	QM7 cultured on corn husk scaffolds yielded increased protein, but PBSCs seeded on corn husks did not yield protein content higher than controls (QM7 on corn husk: 16.28 ± 3.55, PBSCs on corn husks: 9.57 ± 1.56 ug/ul lysate/Gram, control: 6.35 ± 1.43 ug/ul lysate/Gram).	[65]
Rai, 2023	Apple	Chemical: SDS	Infusion with *Lactobacillus* sp. cells	Drug delivery/ Probiotic delivery	Alginate-coated scaffolds aided in the survival of the *Lactobacillus* sp. after gastric- and intestinal-simulated transits.	[66]
Salehani, 2023	Olive Leaves	Chemical: (a)n-Hexane > SDS > NaClO (b) n-Hexane > Triton-X 100 and NaClO	C619	Cytotoxicity	Samples with higher SDS concentrations showed higher cytotoxic effects.	[67]
Xia, 2023	Lotus Petioles	Chemical: SDS > Sodium hypochlorite	hMSC HUVEC hDF hESC	Cell attachment, proliferation, and alignment	Poly-L-lysine, PVG copolymer coating, and peptide (CGGGRGDSP-am (RGD) or CGGGK*(FITC)-am) immobilization on the lotus scaffold. All four cell lines showed polygonal morphologies after incubation for 1 h on to the RGD-decellularized scaffold and no attachment on the PVG- decellularized s ones. After 7 days of culturing, the hESC, hDF, and hMSC align along the plant’s topography.	[68]
Yun, 2023	Sorghum Leaves	Chemical: NaOH > Sodium hypochlorite	hESC	Adhesion, growth, and alignment Myogenic differentiation and contraction	The leaf scaffold was biofunctionalized with poly (PEGMEMA-*r*-VDM-*r*-GMA) copolymer, which prevented non-specific protein adsorption, and was modified with cell adhesive RGD peptide to enable cell adhesion and growth in serum-free media. The hESC-derived myogenic progenitor cells cultured on the biofunctionalized leaf scaffold adopted a parallel orientation along the leaf’s topography and showed uniaxial contraction.	[69]
Zamudio-Ceja and Garcia-Contreras, 2023	Nopal	Chemical: SDS > CaCl_2_	hDPSC	Osteogenic differentiation	hDPSCs showed significant increases in cell viability of 95% and 106% at 168 h for native and decellularized scaffolds, respectively. Cells grown on TCP showed fusiform elongated morphology, and the ones on the decellularized scaffold showed a spherical shape. Also, the scaffold by itself did not induce the pro-inflammatory expressions of COX-1 or COX-2 but permitted physiological cell function under IL-1β stimulation.	[70]
Ahmadian, 2024	Eggplant Leaves	Chemical: SDS > n-Hexane>	HepG2 cells	Tissue/disease model	Model for hepatocarcinoma tissue. The 3D model was assessed by seeding HepG2 cells on decellularized eggplant leaves to check the effect of prilocaine on cancer cells. Evidence suggests that HepG2 cells were able to thrive and proliferate effectively on the scaffold, and prilocaine demonstrated its efficacy in inhibiting the growth of cancer cells. The model successfully mimics the tissue and the drug interactions.	[71]
Banaeyan, 2024	Watermelon Rind	Chemical: SDS	hFF hMSC	Cytotoxicity and metabolic activity, osteogenic differentiation	Polydopamine treated watermelon rind had a higher cell density and aided in the osteoinduction of the hMSCs evidenced by the mineralization, deposition of hydroxyapatite crystals and raised gene expression of COL1A1, BGLAP, ALP, RUNX2 and SPP1 after 21 days of culture. The coated scaffolds showed the greatest calcium deposits and ALP activity when compared to the TCP and uncoated scaffolds.	[72]
Berry-Kilgour, 2024	*Ulva lactuca*, *Ecklonia radiata*, and *Durvillaea poha*	Chemical + Physical: (a) SDS > NaCO_3_ + NaClO + Temperature (b) SDS > Triton X-100 + NaClO	Immortalized dermal fibroblasts (BJ/5Ta)	Cytotoxicity and attachment	No matrix showed cytotoxic effects. When seeded on *D. poha* or *U. lactuca* scaffolds, fibroblasts were rounded with limited cellulose contact. By contrast, fibroblasts attached to the fibrous inner layer of the *E. radiata* scaffolds.	[73]
Cevik, 2024	Parsley	Chemical: SDS > Triton X-100 > Sodium hypochlorite	L929 HUVEC	Cytotoxicity attachment, proliferation, and development of tissue-engineered vascular grafts	Parsley stems were used to produce a biocompatible scaffold for TEVG applications. Cytotoxicity and proliferation assays with L929 cells showed no difference when compared to the TCP control. No short-term and long-term cytotoxicity was found for the decellularized parsley stems. The scaffolds were suitable for the culture of human endothelial cells, where monolayer formation was observed over 7 days.	[74]
Chen, 2024	Parsley	Chemical (a) SDS > Sodium hypochlorite (b) Triton X-100 > Sodium hypochlorite	C2C12	Cultured meat	Plant based scaffolds were modified with type A gelatin and crosslinked with transglutaminase. After induced differentiation, the fibrous scaffolds were more inclined to form multinucleated myotubes with higher expression of myogenic genes and proteins, and the final cell-based meat contained higher total protein content than the honeycomb structure.	[75]
Dikici, 2024	Spinach	Chemical: Acetic acid > SDS > Triton X-100 > Sodium hypochlorite	L929	Drug delivery	Baby spinach leaf scaffolds were loaded with L-ascorbic and then released within the effective dose range. The spinach scaffolds releasing the ascorbic acid showed an increase in cells’ metabolic activity.	[76]
Filiz, 2024	Spinach	Chemical: n-Hexane > SDS > Triton X-100 > Sodium chlorite	MCF-7 and HUVEC, and spheroids. HDFs embedded in GelMA to mimic breast tissue with tumor	Cancer/disease/tissue model	The endothelialization of decellularized spinach without any surface modification was done. The developed micro platform enabled the co-culture of breast cancer spheroids and plant-derived vasculature under perfusion flow, resulting in close-to-real breast cancer modeling.	[77]
Fiorelli, 2024	Celery	Chemical: SDS	hASC	Cell viability and osteochondrogenic differentiation	24h decellularized scaffolds were used. After 3 weeks the cells showed osteogenic differentiation. Comparison with GelMA scaffolds correlates. Higher content of GelMA, indicated greater stiffness, and more osteogenic differentiation. Lesser content, chondrogenic differentiation.	[78]
Gorbenko, 2024	Leatherleaf Viburnum	Chemical: SDS > Triton X-100 and Sodium hypochlorite	rEC Vascular smooth muscle cells Rat citrated blood White cell assay	Cell adhesion, proliferation. In vitro thrombosis. Foreign body reactions in vitro.	Grafts coated with fibronectin were seeded with vascular smooth muscle cells and endothelial cells. Endothelial cell density after 24 h of exposure to fluid flow did not change significantly compared to static culture. Endothelial cell density significantly increased by 30% after 3 weeks of bioreactor treatment with fluid compared to 3 weeks of culture under static conditions. Endothelialization of leatherleaf scaffolds significantly reduces thrombus formation in vitro. The application of fluid flow and pressure can further reduce thrombosis. After 24 h in culture, the greatest white cell density was found in endothelial cell and vascular smooth muscle cell seeded leatherleaf, followed by TCP control and acellular decellularized scaffold.	[79]
Hasanzadeh, 2024	*Beaucarnea recurvata* Leaves	Chemical: n-Hexane > SDS > Triton X-100 + Sodium hypochlorite > Hexane	hDP-SC	Cell attachment, proliferation, osteogenic differentiation	Grape seed proanthocyanidin coated plant-based scaffold had improved physicochemical properties, as well as biological ones such as cell proliferation, protein absorption, osteogenic differentiation. This last one was evident by an increase in ALP activity, and mineral deposition.	[80]
Hong, 2024	Celery	Chemical: SDS > Ethanol	Chicken myoblast	Cultured meat	SDS decellularized celery scaffolds were nontoxic for the cells and supported proliferation and differentiation. After 2 weeks fully grown myoblasts completely covered the surface of the scaffold.	[81]
Hosseini, 2024	Pumpkin	Chemical: NaClO + NaHCO_3_	HFF MG63 Wistar rats hASC	Cytotoxicity, cell adhesion. In vivo biocompatibility and inflammation. In vitro osteoinductivity (osteogenic differentiation).	Both coated with magnesium oxide and uncoated scaffolds did not induce cytotoxicity. For cell adhesion and proliferation in the magnesium oxide coated scaffolds showed a higher percentage of live cells at all time points. In vivo, tests showed no adverse effects on the overall health of the animals. Moreover, the animals with coated scaffolds showed the highest wound closure percentage.	[82]
Hu, 2024	Apple	Chemical: SDS	ASC BALB/c mice	Cell attachment and proliferation. Hepatocyte-like induction. Acute liver injury.	Apple-derived cellulose scaffolds served as successful platforms for the growth and attachment of ASC. Liver function recovered in ALI mice transplanted with the apple decellularized scaffold and the implants developed vasculature and bile duct structures.	[83]
Ksouri, 2024	Spinach	Chemical: SDS > Tween-20 > SDS + Sodium hypochlorite	*S. aureus * NIH3T3 A549 BEAS-2B	Antimicrobial activity. Cell viability, wound healing ability.	Decellularized spinach scaffold showed antibacterial activity. The scaffolds showed no cytotoxicity. Decellularized constructs had the capability to boost the migration of cells, ideal for wound healing applications.	[84]
Singh, 2024	*Ficus benjamina*	Chemical: n-Hexane > SDS > Tri-n-butyl phosphate > Sodium hypochlorite	Rabbit RBC New Zealand White rabbits	In vitro hemocompatibility test. In vivo biocompatibility analysis.	The decellularized scaffolds showed less hemolysis than the native *F. benjamina*. Histological analysis of the implanted scaffolds showed encapsulation of the native tissue indicating a moderate to severe immune response. On the other hand, the decellularized scaffolds showed epidermal cells and less inflammatory infiltrate.	[85]
Leblanc-Latour, 2024	Apple	Chemical: SDS	MC3T3-E1 Sprague-Dawley rats	Cell adhesion, proliferation, osteogenic differentiation. Calvarial defect model	Seeded scaffolds showed mineralized deposits after 4 weeks in culture with differentiation medium. Visual assessment indicated the scaffolds seemed well integrated in the skull surrounding tissues. H&E staining revealed cellular infiltration within the scaffold pores and evidence of vascularization, as shown by the presence of blood vessels within the scaffolds.	[86]
Luo, 2024	Natural Leaf Veins (NLVs)	Chemical + Physical: NaOH + Temperature > H_2_O_2_	PC-SC dASC	Cultured meat	After adhesion, proliferation, and differentiation of the cell lines onto the natural leaf veins scaffolds, muscle and fat slices were produced.	[87]
Raundal, 2024	Spinach	Chemical: Ethanol + NaOH + HCl	hDP-SC	Cell attachment, osteogenic differentiation	Cells were able to attach to the cell surface and penetrate it. On day 14, spinach scaffold had significantly higher osteonectin gene expression than the control.	[88]
Sadegh-Zaman, 2024	*Lisianthus* sp.	Chemical: NaOH + NaOCl as Sodium hypochlorite	Schwann cells	Cell proliferation and migration for neural tissue engineering	To assess this potential, the *Lisianthus* flower stems were decellularized and then modified with polyaniline and graphene oxide nanosheets (0.05% (G0), 0.1% (G1), 0.2% (G2), and 5% *w*/*v* (G3)) and graphene oxide nanosheets. Modified and decellularized stems had no cytotoxicity to the Schwann cells	[89]
Singh, 2024	*Bougainvillea* sp.	Chemical: TRIS-HCl (0.5 M, pH 8.0) > NaOH + SDS > Triton X-100 + Sodium hypochlorite	HaCaT NIH/3T3	Cell proliferation	The bougainvillea scaffold did not show cytotoxicity. The actin morphology of NIH/3T3 cells on the floral scaffold exhibited formation of a well spread filamentous network. The increase in cellular density of HaCaT signifies the preference of the cellular attachment onto the floral scaffold.	[90]
Sood, 2024	Apple	Chemical: n-Hexane > SDS > Triton x-100 + Sodium hypochlorite	PB-SC and in co-culture with NIH/3T3 fibroblasts	Cultured meat	Decellularized apple coated with a polymer mixture of gelatin/alginate. Coated scaffolds showed enhanced capability to adhere and proliferate the two cell lines on their surface, compared to uncoated apple scaffolds. Also coated ones were more easily digested in simulated gastric fluid with pepsin. Both coated and uncoated scaffolds behaved similarly when incubated with simulated gastric fluid and PBS.	[91]
Will, 2024	*Taraxacum Ruderalia*	Chemical: SDS > CaCl_2_	Human and murine skin fibroblasts and dermal lymphatic endothelial cells HDLEC	Biocompatibility, proliferative capacity, and ex-vivo endothelialization	No statistical difference in cell growth was found for HDLEC in-vitro on equivalent sheets of SDS-decellularized cellulose compared to a commercial neo-dermis. The tubes showed adequate biocompatibility, supported cell proliferation, and facilitated spontaneous ex-vivo endothelialization of lymphatic endothelial cells. In the ex vivo swine limb model, Lympho-Venous Anastomoses using the engineered cellulose tubes was successfully performed.	[92]
Yang, 2024	Pomelo	Chemical: SDS > Triton X-100 + Sodium hypochlorite	L929, Raw264.7 HUVECs *S. aureus* + *E. coli* Sprague-Dawley rats	Cytotoxicity In vitro cell migration, angiogenesis test. In vitro antibacterial test In vivo antibacterial and wound healing in infected wounds.	Hybrid wound dressing, with the decellularized pomelo as the substrate material. The PVA-TSPBA hydrogel works as a coating material to enhance the pomelo’s adhesive and moisture retention ability. The gallic acid/Cu MOFs are antibacterial agents. In vitro, the DPP + MOF@Gel exhibits good biocompatibility. Moreover, the DPP + MOF@Gel can inhibit the viability of *S. aureus* and *E. coli* both in vitro and in vivo.	[93]
Imeidopf, 2025	*Leatherleaf viburnum*	Chemical: (a) Trypsin > Tergitol (b) Trypsin > Tergitol > EGTA (c) SDS > Tergitol (d) SDS > clearing/Sodium hypochlorite	rEC	Adhesion and proliferation	Fibronectin coated decellularized scaffolds were seeded with rat aortic EC. Results showed that the best protocol in terms of cell proliferation and survival was SDs with clearance of less than 6 h. Scaffolds treated with Trypsin/Tergitol/EGTA showed the least survival rate. Additionally, for the SDS treated scaffolds, an increment in the clearance time was inversely proportional to the cell density.	[94]
Kian, 2025	Walnut Leaves	Chemical: n-Hexane > SDS > TritonX-100 + sodium chlorite	hMSC Balb/c mice	Cell adhesion and proliferation. Wound closure analysis and histopathological analysis.	The decellularized walnut leaves scaffold is nontoxic and cytocompatible. SEM images show a mixture of circular and elongated morphologies on the surface of the scaffolds. Animal treated with the walnut decellularized scaffolds showed a higher wound closure percentage on days 3 and 14 when compared to controls. Same for histopathological scores and collagen deposition. Wounds treated with the walnut scaffold had better re-epithelialization, collagen deposition, and angiogenesis.	[95]
Lee, 2025	Spinach	Chemical: Acetone > SDS > Triton x-100 > Sodium hypochlorite	None	None	The decellularized spinach scaffold effectively mimics the vasculature architecture. The scaffolds maintain an intact cellulose framework and vein system, as evidenced by the flow of fluorescent molecules and the reversible color changes of adsorbed colorimetric nanoparticles in response to pH variations.	[96]
Salehi, 2025	Water Spinach, Green Onion, and Water Horsetail	Chemical: n-Hexane > SDS > Triton X-100 + Sodium hypochlorite	HUVEC	Cell adhesion, vitality, and proliferation	HUVECs on the luminal surfaces of decellularized scaffolds show higher expression of Ki-67 protein and a consistent increase in cell number on water spinach and green onion scaffolds compared to TCP.	[97]
Yang, 2025	Shiitake Mushroom, Oyster Mushroom, King Oyster Mushroom, and Wood Ear Mushroom	Chemical: SDS	C2C12	Cultured meat	Shiitake, Oyster, and Wood ear mushroom microcarriers showed 92.7%, 90%, and 62.7% and of initial attachment of animal cells, respectively. King Oyster mushrooms samples failed to clear the remnants of the SDS, so there was no cell survival. There was no significant difference in proliferation between the Shiitake mushroom microcarrier and the TCP control.	[98]

**Table 3:** Most frequently used chemicals for plant-based matrix decellularization and their reported LD50 values [100].

Chemical	Inhibitory Concentration 50× (mM)	Oral Rat or Mouse LD50 (mmol/kg)	Oral Rat or Mouse LD50 (mg/kg)
Acetone	444	168	9759.1
Ethanol	379	304	14,008.3
Sodium chloride	75.9	51.3	2998
Sodium dodecyl sulfate	0.27	4.45	1288
Triton X-100	0.055	2.78	1798.7
Tween 80	0.49	19.1	25,021

## Abbreviations

ALP	Alkaline Phosphatase
APTES	Amino (NH2)-Terminated 3-Aminopropyltriethoxysilane
RGP peptide	Arginine–Glycine–Aspartate Peptide sequence commonly found in ECM Adhesion Proteins
AFM	Atomic Force Microscopy
BET	Brunauer–Emmett–Teller
CaCl2	Calcium Chloride
ciPTEC	Conditionally Immortalized Proximal Tubule Epithelial Cell
dASC	Dog-Adipose-Derived Stromal Cell
EGTA	Ethylene Glycol-Bis (Β-Aminoethyl Ether)-N, N,N′,N′-Tetraacetic Acid
ECM	Extracellular Matrix
FTIR	Fourier-Transform Infrared Analysis
GO	Graphene Oxide
THP-1	Human Acute Monocytic Leukemia
A549	Human Adenocarcinoma Alveolar Basal Epithelial Cell
hASC	Human-Adipose-Derived Stem Cell
hBM-MSC	Human-Bone-Marrow-Derived Mesenchymal Stem Cell
MCF-7	Human Breast Adenocarcinoma Cell
MDA-MB321	Human Breast Cancer Cell Line Expressing GFP
hDP-SC	Human-Dental-Pulp-Derived Stem Cell
hDF	Human Dermal Fibroblast
HDLECs	Human Dermal Lymphatic endothelial cells
hESC	Human Embryonic Stem Cell
hEC	Human Endothelial Cell
HeLa	Human Epithelial Cells Derived from Cervical Cancer
hFFs	Human Foreskin Fibroblasts
HepG2	Human Hepatocarcinoma
BJ/5Ta	Human-Immortalized Dermal Fibroblast
HaCaTs	Human-Immortalized Keratinocytes
hiPS-CMs	Human-Induced Pluripotent-Stem-Cell-Derived Cardiomyocytes
hiPSC	Human-Induced Pluripotent Stem Cell
Lin Sca-1pos cMSCs	Human Lin Sca-1pos Cardiac Mesenchymal Cells (CMSC)
BEAS-2B	Human Lung Epithelial Cell Line
hMSC	Human Mesenchymal Stem Cell
MG63	Human Osteosarcoma Cell Line
hPS-CMs	Human Pluripotent Stem-Cell-Derived Cardiomyocytes
PC3	Human Prostate Cancer Cell
hRBC	Human Red Blood Cell
hSMC	Human Skeletal Muscle Cell
HUVEC	Human Umbilical Vein Epithelial Cell
HCl	Hydrochloric Acid
IVC	Inferior Vena Cava
A549	Lung Adenocarcinoma Cell Line
MOFs	Metal-Organic Frameworks
OTS	Methyl (CH3)-Terminated Octadecyltrichlorosilane
C619	Mouse-Derived Epithelial Cell Line from Skin BALB/C
3T3-L1	Mouse Embryonic Pre-Adipocytes
MC-3T3-E1	Mouse Embryonic Pre-Osteoblasts
NIH-3T3	Mouse Fibroblast
L929	Mouse Fibroblast Cell Line
C2C12	Mouse Muscle Myoblast
MC-3T3	Mouse Pre-Osteoblastic Cell Line
NHDF	Normal Human Dermal Fibroblast
PRP	Platelet-Rich Plasma
PLGA	Polylactic-Co-Glycolic Acid
PB-SC	Primary Bovine Satellite Cell
PC-SC	Primary Canine Satellite Cell
QM7	Quail Muscle Clone 7 Cell Line
rASC	Rat-Adipose-Derived Stem Cell
rAEC	Rat Aortic Endothelial Cell
rBMSC	Rat Bone Marrow Stromal Cell
PC12	Rat Cell Line derived from a pheochromocytoma of the adrenal medulla, which has an embryonic origin in the neural crest
rEC	Rat Aortic Endothelial Cell
rH-NSC	Rat Hippocampal Neural Stem Cell
rASCs	Rat Mesenchymal Stem Cells from Adipose Tissue
rVSMC	Rat Vascular Smooth Muscle Cell
SEM	Scanning Electron Microscopy
SK-MEL-28	Skin Melanoma Cell
NaOH	Sodium Hydroxide
TGA	Thermogravimetric Analysis
TCP	Tissue Culture Plate
XRD	X-Ray Diffraction
